# Low Genetic Diversity and Complex Population Structure in Black Piranha ( *Serrasalmus rhombeus* ), a Key Amazonian Predator

**DOI:** 10.1002/ece3.70824

**Published:** 2025-02-17

**Authors:** Alizée Thomas, François‐Étienne Sylvain, Eric Normandeau, Nicolas Leroux, Aleicia Holland, Adalberto Luis Val, Nicolas Derome

**Affiliations:** ^1^ Institut de Biologie Intégrative et Des Systèmes Université Laval Québec City Quebec Canada; ^2^ Fisheries and Oceans Gulf Fisheries Center Moncton New Brunswick Canada; ^3^ Plateforme de Bio‐Informatique de l'IBIS (Institut de Biologie Intégrative et Des Systèmes) Université Laval Québec Canada; ^4^ Department of Ecology, Environment and Evolution, School of Life Science La Trobe University Bundoora Victoria Australia; ^5^ Laboratório de Ecofisiologia e Evolução Molecular Instituto Nacional de Pesquisas da Amazônia (INPA) Manaus Brazil

**Keywords:** Amazon, black piranha, cryptic species, genetic diversity, population dynamics

## Abstract

The black piranha ( *Serrasalmus rhombeus* ), a widely spread species in the rivers of the Amazon basin, plays a vital role as both key predator and important prey. Despite its essential contribution to ecosystem stability, there is a lack of information regarding its genetic diversity and population dynamics in the central Amazon region. As the Amazon continues to undergo environmental changes in the context of growing anthropogenic threats, such knowledge is fundamental for assist in the conservation of this species. This study is the first to analyze the genetic diversity and population structure of *S. rhombeus* in the central Amazon region using high‐resolution genomic data. We employed a Genotyping‐by‐Sequencing approach with 248 samples across 14 study sites from various tributaries, encompassing diverse water types (black, white, and clear water) and characterized by 34 physiochemical parameters. The data reveals low diversity accompanied by pronounced signs of inbreeding in half of the sites and robust genetic differentiation and variation among sites and within‐sites. Surprisingly, we also found evidence of higher dispersal capacity than previously recognized. Our analysis exposed a complex and high population structure with genetic groups exclusive to some sites. Gene flow was low and some groups presented ambiguous genealogical divergence index (*gdi*) signals, suggesting the occurrence of potential cryptic species. Moreover, our results suggest that the population structure of black piranha appears more influenced by historical events than contemporary factors. These results underscore the need to give greater attention to this keystone species, for which no regulatory framework or conservation strategies is presently in effect.

## Graphical abstract

Using a Genotyping‐by‐Sequencing approach across tributaries in the central Amazonia, we found that the black piranha ( *Serrasalmus rhombeus* ) exhibits low genetic diversity, pronounced inbreeding, and significant population structure, likely shaped by historical events rather than contemporary factors.

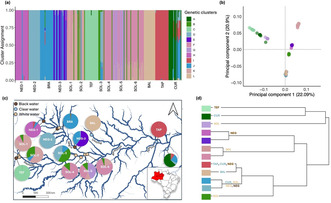

## 1 Introduction

The black piranha ( *Serrasalmus rhombeus* ) holds a pivotal role in the Amazon ecosystem, serving as predator, prey, and even seed disperser (Goulding 1980; Loiselle et al. 2001; Weiss et al. 2024). Beyond its crucial contribution to maintaining ecological balance, *S. rhombeus* represents a valuable source of proteins and commerce for *ribeirinho* communities (Silva and Begossi 2013) and a popular target in sport fisheries (Begossi et al. 2018; Santos, Ferreira, and Zuanon 2009). This charismatic species is the subject of popular touristic activities, including fishing and sale of ornamental souvenirs (D'Cruze et al. 2017). Despite its significance, there is a notable lack of comprehensive information regarding the distribution, abundance, and dynamics of *S. rhombeus* populations. This knowledge gap poses a challenge in formulating effective conservation and management strategies for this ecologically significant species. The Amazon waters are experiencing significant environmental changes, including more intense floods, droughts, higher temperatures, increased water acidity, and more hypoxic episodes (Val and Wood 2022). These waters are also threatened by human activities such as deforestation, dam construction, pollution, silting, oil extraction, and mining (Azevedo‐Santos et al. 2021; Pelicice et al. 2021; Piorski et al. 2008). In addition, predators like *S. rhombeus* are more exposed to pollutants through biomagnification (Kelly et al. 2007; de Queiroz et al. 2021). The capacity of black piranha to negotiate these threats will depend on the resilience potential of populations from this species. Population resilience is in turn heavily dependent on genetic diversity, which fuels adaptability by increasing the likelihood of expressing advantageous traits for thriving in changing environments. Consequently, populations exhibiting higher genetic diversity are more likely to harbor individuals with beneficial variations conducive to environmental changes (Hughes, Daily, and Ehrlich 1997; Hughes et al. 2008).

Ecological and geographical variables play important roles in shaping population structure and diversity by influencing the transfer of genetic material from one population to another within a species (Neelabh. 2022). First, gene flow can be reduced by distance‐limited dispersal, leading to isolation‐by‐distance (IBD). Therefore, gene flow is inversely proportional to the distance between geographical sites (Sexton, Hangartner, and Hoffmann 2014). Second, river water flow can influence site connectivity, as currents may act as a physical barrier by hindering upstream movement and limiting the exchange of genetic material between populations (Delord et al. 2023; Leroux et al. 2022). Third, contrasting environments in the Amazon, such as black water (high DOC, low pH 3.5–6, low nutrients, low conductivity 5–20 μS cm^−1^), clear water (pH 5.5–8, low nutrients, low conductivity 5–40 μS cm^−1^), and white water (pH 6.5–7, higher conductivity 40–300 μS cm^−1^, nutrient‐rich, and turbid) (Bogotá‐Gregory et al. 2020; Goulding, Carvalho, and Ferreira 1989; Junk and Piedade 2011; Ríos‐Villamizar et al. 2013; Sioli 1984; Val and de Almeida‐Val 1995), can limit gene flow between populations, depending of the species ecology and sensitivity to those parameters. Indeed, fish individuals adapted to a given water type would likely have a lower fitness in a different water type (Isolation‐By‐Environment [IBE]) (Cooke, Chao, and Beheregaray 2012a, 2012b, 2012c; Cooke, Landguth, and Beheregaray 2014). Fourth, population gene flow and structure can be influenced by historical processes, such as uplift of structural arches, marine incursion, and regressions (Lundberg et al. 1998; Piorski et al. 2008; Sexton, Hangartner, and Hoffmann 2014; Val et al. 2021). Indeed, such climatic shifts or geological events can alter aquatic habitats, leading to isolation or mixing of populations, leaving a lasting imprint for millennia (Avise 2000). It has been suggested that predators that hunt by sight such as *S. rhombeus* are better adapted to clear water (black and clear water) than turbid water (white water) (Saint‐Paul et al. 2000; Santos, Ferreira, and Zuanon 2009). However, a study carried out on *S. rhombeus* in the upper Rio Madeira (Bolivia) did not find any effect of water type on the strong genetic clustering observed, but did find an effect of geographical distance and historical events (Hubert et al. 2007a). Another study, conducted in the Maroni River (French Guiana), showed an effect of site connectivity by continuous, unidirectional water flow rather than geographical distance (Delord et al. 2023). Those different findings highlight the difference of IBD effect depending on river connectivity. The mechanisms influencing gene flow for *S. rhombeus* are therefore not fully understood and require additional investigation.

Isolation between populations can lead to the accumulation of genetic differences that could eventually lead to speciation. In fact, *S. rhombeus'* taxonomy has been widely debated. In his work on the family Serrasalmidae, Géry (1976) suggested that *S. rhombeus* could be considered a complex of six to nine species. More recent studies conducted on karyotypes (Nakayama et al. 2001; Nakayama, Feldberg, and Bertollo 2012; Teixeira et al. 2006), isoenzymes (Teixeira et al. 2006), as well as subtle morphological differences such as size and gill parasite community (Every and Kritsky 1992; Nakayama et al. 2001) have suggested the presence of cryptic species among black piranha. Species are considered cryptic when they are classified as the same species based on morphological similarities when in fact, they represent different species (Bickford et al. 2007) that are distinct in their genetic lineages (Tan et al. 2010). The development of molecular tools has made it possible to study genetic divergence between different groups and has thus made a major contribution to species delimitation, that is, identifying biological diversity at the species level (Carstens et al. 2013). In many cases, only a few loci are used, particularly mitochondrial DNA due to its high mutation rate (Griffiths et al. 2010; Marzouk et al. 2017; Melo et al. 2016; Piggott, Chao, and Beheregaray 2011; Shirley et al. 2014). However, for recent speciation, as would be the case for *S. rhombeus* (Machado et al. 2018; Teixeira et al. 2006), the use of a small number of loci often proves insufficient (Moritz and Cicero 2004; Weiss et al. 2018), as shown in studies on *S. rhombeus* using mitochondrial DNA (Machado et al. 2018), 18S and 5S rDNA (Nakayama, Feldberg, and Bertollo 2012), and introns (Hubert et al. 2006). Approaches like Genotyping‐By‐Sequencing (GBS) produce thousands or tens of thousands of SNP variants that give access to more accurate species‐level information (Daïnou et al. 2016; Shaffer and Thomson 2007). This can lead to the discovery of cryptic species that were left undetected by the standard mitochondrial DNA approaches (Wang et al. 2020; Weiss et al. 2018).

In order to enhance our knowledge about the genetic diversity and distribution patterns of the black piranha ( *S. rhombeus* ), we aimed to (1) define population structure of *S. rhombeus* in the central Amazon, providing insights into the evolutionary forces shaping it and (2) investigate the genetic signature of putative cryptic species within this clade to then open the door for further research in the region with a more integrative approach to confirm their occurrence. In order to address these questions, we used GBS, a method that allowed us to generate genome‐wide single nucleotide polymorphism (SNP) markers (Elshire et al. 2011). Overall, we sampled 248 individuals from 14 different sites comprising five watersheds of the Amazon and three different water types (black, white, and clear water) and characterized by 34 different physicochemical parameters.

Our hypotheses are: (i) black piranha populations exhibit high genetic structure due to limited gene flow; (ii) this genetic structure is primarily influenced by geographical isolation, where greater distances between sites lead to reduced gene flow (Hubert et al. 2007a), and by site connectivity, where water currents hinder upstream movement (Delord et al. 2023), rather than differences in water type, as variations in physicochemical parameters between sites would not impede individual movement (Hubert et al. 2007a); and (iii) some of these genetic groups may represent cryptic species (Nakayama et al. 2001; Nakayama, Feldberg, and Bertollo 2012; Teixeira et al. 2006).

## 2 Material and Methods

### 2.1 Sampling

**FIGURE 1:**
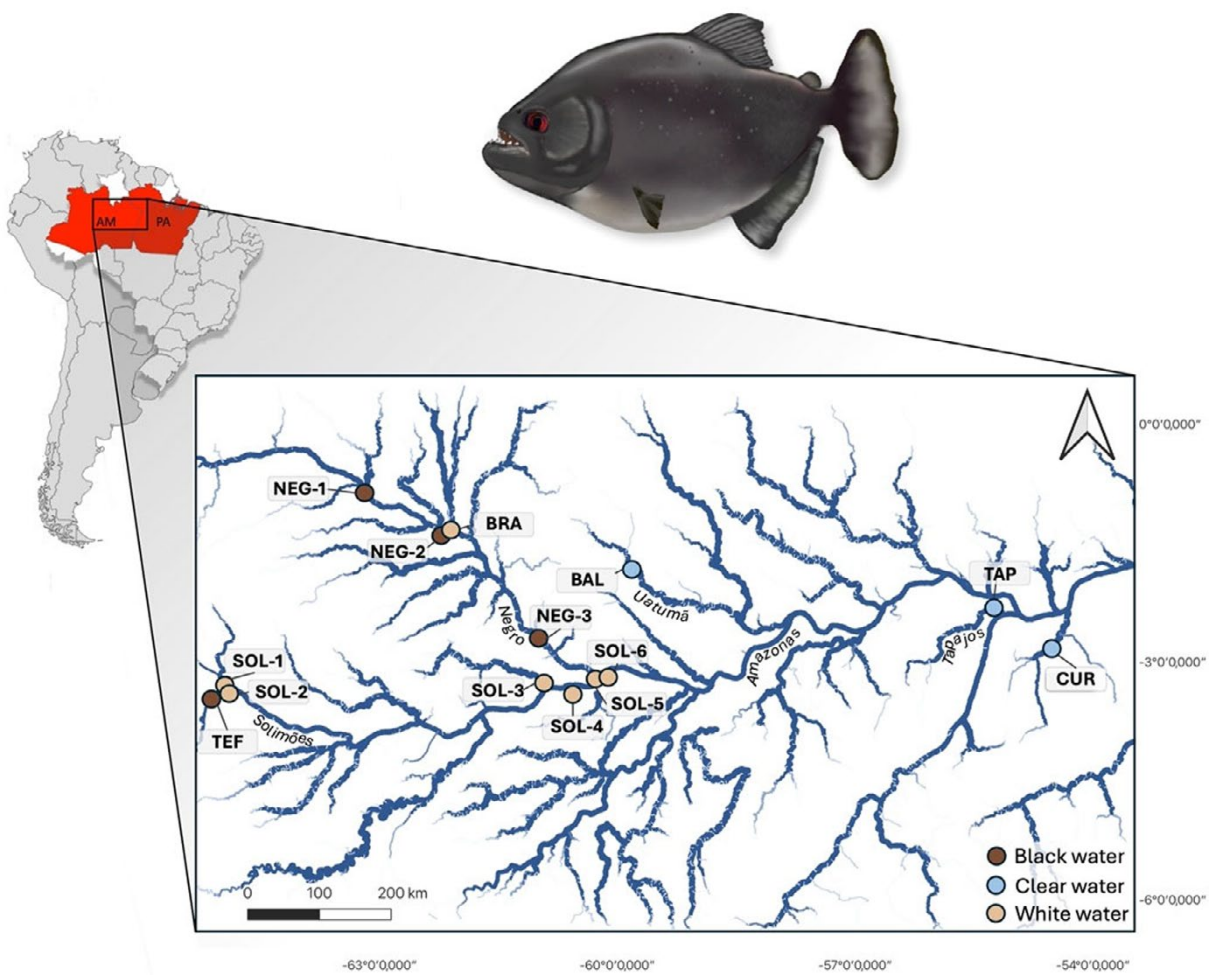
Map of sampling sites, sampled in 2018 and 2019 in the Amazon. The map was made with QGIS software version 3.24.3 with the GPS coordinates of the sampling sites (Table S1) and hydrographic data from the *catálogo de metadados da Agência Nacional de Águas—Instituto Brasileiro de Geografia e Estatística*. Illustration of black piranha ( *Serrasalmus rhombeus* ) by Alizée Thomas.

A total of 248 individuals of *S. rhombeus* were collected in the Amazon Basin (Amazonas and Pará states, Brazil) in 2018 and 2019 during the dry season (Table S1) at 14 sites in different Amazon River tributaries, including seven white water, four black water, and three clear water sites (Figure 1). Fin clip samples were collected and immediately preserved in nucleic acid preservation (NAP) buffer to preserve maximum DNA integrity (Camacho‐Sanchez et al. 2013). The samples were then stored at −20°C. In addition, shortly after conducting fish sampling, a sample of environmental water was collected and 34 physicochemical parameters were measured (Tables S2–S5) (detailed methodology in Sylvain et al. 2023). The fieldwork was done under the permits SISBIO 29837‐12 and CPAUL 2018021‐1. Sample Exportation was done under the permit 00000481/2028‐UVGAMAO‐AM.

### 2.2 DNA Extraction

We first dissected the fin tissue (20 mg) and performed DNA extraction using the *QIAGEN DNeasy Blood and Tissue* kit according to the manufacturer's instructions, without modification, at the *Instituto Nacional de Pesquisa da Amazonia* (INPA). The resulting DNA solutions were then analyzed spectrophotometrically using a Nanodrop instrument to measure nucleic acid concentration and quality. DNA solutions were preserved at −20°C.

### 2.3 GBS

Preparation of the GBS library was made following the protocol of Abed et al. (2019). In brief, DNA was first digested with *Cutsmart buffer* and with *PstI* and *MstI* restriction enzymes. Then, the ligation step was done with T4 DNA Ligase reaction buffer. The samples were combined and purified using the *QIAquick PCR Purification kit* (Qiagen, Valencia, CA). The DNA fragments were amplified by PCR, using primers (a) 5′‐CCATCTCATCCCTGCGTGTCTCCGACTCAG and (b) 5′‐CCACTACGCCTCCGCTTTCCTCTCTATGGGCAGTCGGTGAT. The PCR reaction was carried out under the following conditions: an initial denaturation step at 75°C for 5 min, followed by 5 cycles of 10 s at 98°C, 30 s at 50°C, 30 s at 72°C, followed by 7 cycles of 10 s at 98°C, 30 s at 65°C, and 30 s at 72°C; followed by a final elongation of 5 min at 72°C. Samples were then preserved at 4°C.

The amplified DNA fragments were then purified using *Axygen magnetic beads* (Beckman Coulter Genomics). *BioAnalys*er was used to assess library quality. Libraries were sequenced using *the Ion Proton sequencer* from *Ion Torrent* (Thermo Fisher Scientific, USA) at the Plateforme d'Analyses Génomiques from the Institut de Biologie Intégrative et des Systèmes (IBIS) at Laval University.

### 2.4 SNPs Calling

Data preparation, genotyping, and filtration were processed using STACKS v2.62 (https://catchenlab.life.illinois.edu/stacks/) and stacks_workflow v2.62 (https://github.com/enormandeau/stacks_workflow). All filtration scripts used are available at the above link. Briefly, cutadapt v1.18 (‐e 0.2 ‐m 50) was used to trim reads for quality. Samples were demultiplexed with process_radtags v2.62 (‐c ‐r ‐t 80 ‐q ‐s 0 ‐‐barcode_dist_1 2 ‐E phred33 ‐‐renz_1 pstI ‐‐renz_2 mspI). Samples then had an average coverage of 2.41 million reads. We then ran ustacks (‐m 3 ‐M 3 ‐N 5 ‐H ‐‐deleverage), cstacks (‐n 1), sstacks, tsv2bam, gstacks, and population (‐p 2 ‐r 0.5 ‐‐fasta‐loci ‐‐vcf) to produce a minimally filtered VCF file containing 252 samples, 250,182 SNPs, an average genotype coverage of 17.2, and an overall missing genotype rate of 39.77%. After tests on dozens of datasets, the values used for the parameters in ustacks and cstacks (n, m, M, N) were found to work well in a wide variety of fish species and even distant taxa. After these STACKS steps, SNPs were processed as follows. First, SNPs were filtered (05_filter_vcf_fast.py, params: 3 70 0 3) so that all the genotypes had a minimum coverage of three and kept only SNPs for which all the sample groups had at most 30% of missing data and for which at least three samples had the rare allele. This dataset was used to get the proportion of missing data per sample. Samples with more than 15% of missing data (four samples) were excluded from the original VCF form populations.snps.vcf file. This new file was filtered similarly to the first (05_filter_vcf_fast.py, params: 3 60 0 3), so that all the genotypes had a minimum coverage of three and we kept only SNPs for which all the sample groups had at most 40% of missing data and for which at least three samples had a copy of the rare allele. We also visualized the distribution of heterozygosity among the sample groups. The groups showed a range of heterozygosity averages. In the absence of an objective threshold to use for all the groups, no sample was removed based on heterozygosity. Using a modified HD plot approach, we then removed SNPs that displayed signs of paralogy or over‐merging using the following parameters: allele coverage ratio, proportion of heterozygotes, proportion of rare‐allele homozygotes, inbreeding coefficient (*F*_IS_) (scripts 08, 09, and 10 from stacks_workflow). Keeping only canonical SNPs, we finally removed SNPs that were in linkage disequilibrium within each locus using the 11_extract_unlinked_snps.py script from stacks_workflow. SNPs under LD were identified using the 11_extract_unlinked_snps.py script from stacks_workflow. Namely, when two SNPs within a single locus have above 50% of identical genotypes for the subset of samples that possess the rare allele in either of the SNPs, only the first one is retained. In cases of linkage, we kept only one of the linked SNPs. The resulting VCF contained 248 samples, 29,587 SNPs, an average genotype coverage of 18.37, and an overall missing genotype rate of 10.16%.

### 2.5 Population Statistics

We calculated the mean observed heterozygosity (*H*_O_), mean expected heterozygosity (*H*_S_), overall genetic diversity (*H*_T_) among populations, mean fixation index (*F*_ST_), and mean inbreeding coefficient (*F*_IS_) (Wright 1949). We also calculated the corrected overall genetic diversity (*H*_TP_) and corrected fixation index (*F*_STP_). The corrected values consider potential biases such as unequal population sizes and correct measures of diversity or differentiation accordingly. The *F*_IT_ value was obtained according to the relationship *F*_IT_ = (*H*_T_ − *H*_O_)/*H*_T_. The π (pi) value corresponds to nucleotide diversity assessed from pairwise sample distance matrices. We also derived *H*_O_, *H*_S_, and *F*_IS_ values for each site. Values were obtained with the R *package Hierfstat* (v0.5‐11; Goudet and Jombart 2022) with 1000 iterations. Contemporary gene flow was calculated with the formula Nm = [(1/*F*_st_) − 1]/4. (Wright 1931). We also checked the proportion of loci outside Hardy–Weinberg equilibrium (HWE) within populations with the R package *poppr* (v 2.9.4; Kamvar, Brooks, and Grünwald 2015) using Monte Carlo permutations (1000 iterations).

### 2.6 Population Structure

Population structure was explored using ADMIXTURE v1.3.0 (Alexander, Novembre, and Lange 2009), where a Markov Chain Monte Carlo (MCMC) search strategy simultaneously estimates allele frequency and population of origin for each number of *k* putative populations. We determined the number of genetic clusters *k* (groups of individuals of a species that are genetically closer to each other) according to the value of cross‐validation (cv) error tests, with k values ranging from 1 to 20. We selected the lowest cv value, as the cv error measures the degree of generalization of the model to novel data or populations. A lower cross‐validation error indicates a better‐performing model with good predictive capacity. *F*_ST_ between genetics groups were also obtained with ADMIXTURE and we calculated Nm value with the same formula previously mentioned. To visualize relationships between genetic clusters, we also performed a Principal Component Analysis (PCA). The PCA were produced with *Plink* (v1.9; Chang et al. 2015) with the function ‐‐pca. The *egein* values generated were then plotted on R with the *ggplot2* package (v3.5.4; Wickham 2016).

### 2.7 Phylogenetics

We conducted a maximum likelihood (ML) phylogenetic tree with the software IQ‐tree v1.6.12 (Minh et al. 2020). *Bayesian Information Criterion* (BIC) was used to select the best model, using the MF option in “‐m” with 1000 bootstrap steps. We selected the model with the lowest BIC value, “TVM F R5”, and added the function “ASC” to ascertain bias correction. The maximum likelihood (ML) phylogenetic tree was visualized using FigTree software v.1.4.4 (http://tree.bio.ed.ac.uk/software/figtree/).

### 2.8 Effect of Physical and Environmental Parameters on Gene Flow

The effects of geographic distance (km), environmental distance, water type and site connectivity by river water current on genetic distance between sites (*F*_ST_/(1 − *F*_ST_)) were studied with the mantel test implemented in the software *ZT* (Bonnet and de Peer 2002) to detect the correlations between matrices. For the environmental distance, we first selected the variables that did not strongly covariate together (*r* < 0.7) among the set of 34 physicochemical parameters measured. We ended up with pH, Fe Conductivity, Nitrite, Nitrate, Silicate, Chl.a, O2, AL, V, Mn Co, Pb, and Zn. Then, the values of these variables were centralized and normalized with the R package *Vegan* (v2.6‐8; Oksanen et al. 2024). We also generated a genetic distance/geographic distance ratio to detect putative genetic barriers. In addition, we selected groups within the Rio Solimões and Rio Negro to compare their genetic distance/km ratio to assess the effect of the water flow between the two rivers on genetic differentiation. We performed a Wilcoxon–Mann–Whitney test to compare the mean of the two groups. To evaluate whether individuals were grouped by watershed or water type, we used a principal component analysis (PCA) for visualization, followed by a PERMANOVA, performed using the *Vegan* R package (v2.6‐8; Oksanen et al. 2024). We also conducted a Betadisper analysis to verify to what extent the points were equally dispersed around their respective centroids, as it could severely impact interpretation of PERMANOVA results. To further explore the grouping of genetic clusters by water type and tributary, we visualized the *F*_ST_ results between genetic groups by plotting a dendrogram using the R package *ape* (v5.7‐1; Paradis and Schliep 2019). The degree of population structure explained by watershed or water type was studied with an Analysis of Molecular Variance (AMOVA). Two AMOVAs were performed. The first AMOVA was performed on water types, that is, black (NEG‐1, NEG‐2, NEG‐3, TEF) white (BRA, SOL‐1, SOL‐2, SOL‐3, SOL‐4, SOL‐5, SOL‐6), clear (BAL, TAP, CUR). The second AMOVA was carried out on the watersheds Rio Negro (N1, N2, N3, B1) and Rio Solimões (SOL‐1, SOL‐2, SOL‐3, SOL‐4, SOL‐5, SOL‐6, TEF. Watersheds having only one site were discarded from this analysis. This analysis was carried out in R studio using the *poppr* R package (v 2.9.4; Kamvar, Brooks, and Grünwald 2015). The same R package was used to quantify private SNPs per site.

In addition, we calculated Asymmetric Eigenvector Maps (AEM) (Blanchet et al. 2011) and distance‐based Moran's Eigenvector Maps (dbMEM) (Dray, Legendre, and Peres‐Neto 2006), to represent, respectively, water flow connection and spatial distance processes on genetic differentiation. These analyses give information on different spatial scales and provide better information on fine‐scale spatial structures (Diniz‐Filho et al. 2013; Dray et al. 2012). dbMEMs were constructed based on the distance between sites with the R package *adespatia*l (v0.3‐24; Dray et al. 2024) and only positive eigenvalues were kept (Dray, Legendre, and Peres‐Neto 2006). For the AEMs, we created a site‐by‐edge matrix based on river connections and flow direction. This matrix contained the relationships between nodes (i.e., sites) and edges (i.e., connectivity links), following the methodologies described by Blanchet, Legendre, and Borcard (2008) and Parreira, Tessarolo, and Nabout (2023). For both AEMs and dbMEMs, smaller serial numbers (e.g., AEM1, MEM1) represented large spatial scales, and larger serial numbers (e.g., AEM13, MEM13) represented smaller spatial scales. We then performed full distance‐based redundancy analysis (db‐RDA) and partial db‐RDA with those variables. We used the variance inflation factor (VIF) with a threshold of 10 to avoid multicollinearity among variables. The most important explanatory AEMs and dbMEMs were selected using a forward selection procedure based on adjusted R^2^ values employing the R package vegan (v2.6‐8; Oksanen et al. 2024) with 1000 permutations. We then used analysis of variance (ANOVA) with 1000 permutations to assess the significance of each variable selected by the model.

### 2.9 Species Delimitation

To characterize the genetic divergence between the previously defined genetic groups and determine the different putative species among the populations, we used the *genealogical divergence index* (*gdi*) (Jackson et al. 2017). This index identifies putative species corresponding to divergence at the species level (Chan and Grismer 2019; Leaché et al. 2019) and has already been used to reveal cryptic species (Chan et al. 2020; Huang 2021; Liu et al. 2019; Poelstra et al. 2021). The *gdi* values range from 0 to 1, where a value < 0.2 means that the groups belong to the same species, a value > 0.7 means that these two groups are two different species and a value between 0.2 and 0.7 shows an ambiguous status. The *gdi* is calculated with the following formula:

$$\mathrm{gdi}=1-e^{-2\;\tau /\theta }$$

where *τ* (tau) corresponds to the divergence time between two populations, and *θ* (theta) is the product of the effective population size (*N*) and the mutation rate per site (*μ*) according to the formula *θ* = 4*Nμ*. In order to calculate the *gdi*, we used the Bayesian Phylogenetics and Phylogeography (BPP) software to conduct A11 analyses to select the phylogenetic tree and A00 analysis to obtain the values needed to calculate the *gdi*. We selected cryptic species candidate groups according to the results of population structure established by ADMIXTURE software, Principal Component Analyses, *F*_ST_ values obtained and maximum likelihood phylogenetic tree. For computational reasons, we performed our analyses on a randomly selected subset of 700 SNPs (Noguerales, Cordero, and Ortego 2018; Meza‐Lázaro et al. 2022) and selected a subset of 70 individuals (*n* = 10 in each group). To do this, we randomly selected individuals in each group. Prior to that, we tested whether there was a difference in *gdi* values (calculated from the results of A00 analyses) between different random selections of 700 SNPs. In our tests, we also performed analyses with 100, 700, and 1000 SNPs and compared the results. We also tested whether there was a difference in *gdi* values between different replicates of SNPs. We also ran tests with different priors for theta and tau (1.10 and 1.10; 2.200 and 2.2000). As done by Huang (2021), we also used the values generated by the program “Minimalist bpp” (https://brannala.github.io/bpps/#/CtrlFile) which generated the values 2, 0.12 and 3, 0.45. Thus, the A00 analyses were then performed with the highest probability score tree among those proposed, and with θ and τ values from the program “Minimalist bpp.” Analyses were performed in triplicates by “backtracking” the tree used (Leaché et al. 2019) to get putative species groups composed of different groups.

## 3 Results

### 3.1 Genetic Diversity

The SNP calling procedure provided a total of 29,587 SNPs. Overall, the genetic diversity was low, with a mean expected heterozygosity (*H*_S_) of 0.11, observed heterozygosity of 0.07, an overall genetic diversity (*H*_T_) and corrected overall genetic diversity (HTP) of 0.15. In addition, the inbreeding coefficient (*F*_IS_) and the mean inbreeding coefficient of an individual relative to the total population were high (*F*_IS_ = 0.23, *F*_IT_ = 0.53).

More specifically, when looking at each site, *H*_S_ values ranged from 0.042 (NEG‐2) to 0.134 (SOL‐5). On the other hand, *H*_O_ values, equally low, ranged from 0.043 (NEG‐2) to 0.101 (SOL‐6). The *F*_IS_ value ranged from −0.038 (NEG‐2) to 0.501 (SOL‐5) (Table 1). NEG‐2 was the only one with negative values. The percentage of loci deviating from Hardy–Weinberg equilibrium varied from 0.406% (TEF) to 17.775% (CUR). Moreover, the number of private SNPs showed a high variation according to the sites and varied from 0 (SOL‐3) to 16.939 (SOL‐5).

**TABLE 1:** Values for expected heterozygosity (*H*_S_), observed heterozygosity (*H*_O_), and inbreeding coefficient (*F*_IS_) for each site.

Site	*H*_S_	*H*_O_	*F*_is_	% OHWE	PSNP
BAL	0.077	0.075	0.008	0.757	1005
BRA	0.081	0.072	0.088	2.342	83
CUR	0.110	0.054	0.488	11.860	12,129
NEG‐1	0.069	0.051	0.255	7.591	712
NEG‐2	0.042	0.043	−0.038	0.670	30
NEG‐3	0.112	0.065	0.421	10.180	370
SOL‐1	0.107	0.096	0.002	3.931	609
SOL‐2	0.130	0.084	0.347	7.649	12
SOL‐3	0.112	0.080	0.288	1.910	0
SOL‐4	0.117	0.096	0.172	4.377	30
SOL‐5	0.135	0.068	0.501	17.775	16,939
SOL‐6	0.110	0.101	0.077	3.660	51
TAP	0.085	0.081	0.027	1.112	382
TEF	0.076	0.075	0.092	0.406	136

*Note:* A positive *F*_IS_ value indicates *inbreeding*, while a value below 0 indicates *outbreeding*. % OHWE represents the proportion of loci outside Hardy–Weinberg equilibrium. PSNP corresponds to the private SNPs for each site.

### 3.2 Site Differentiation and Gene Flow

**FIGURE 2:**
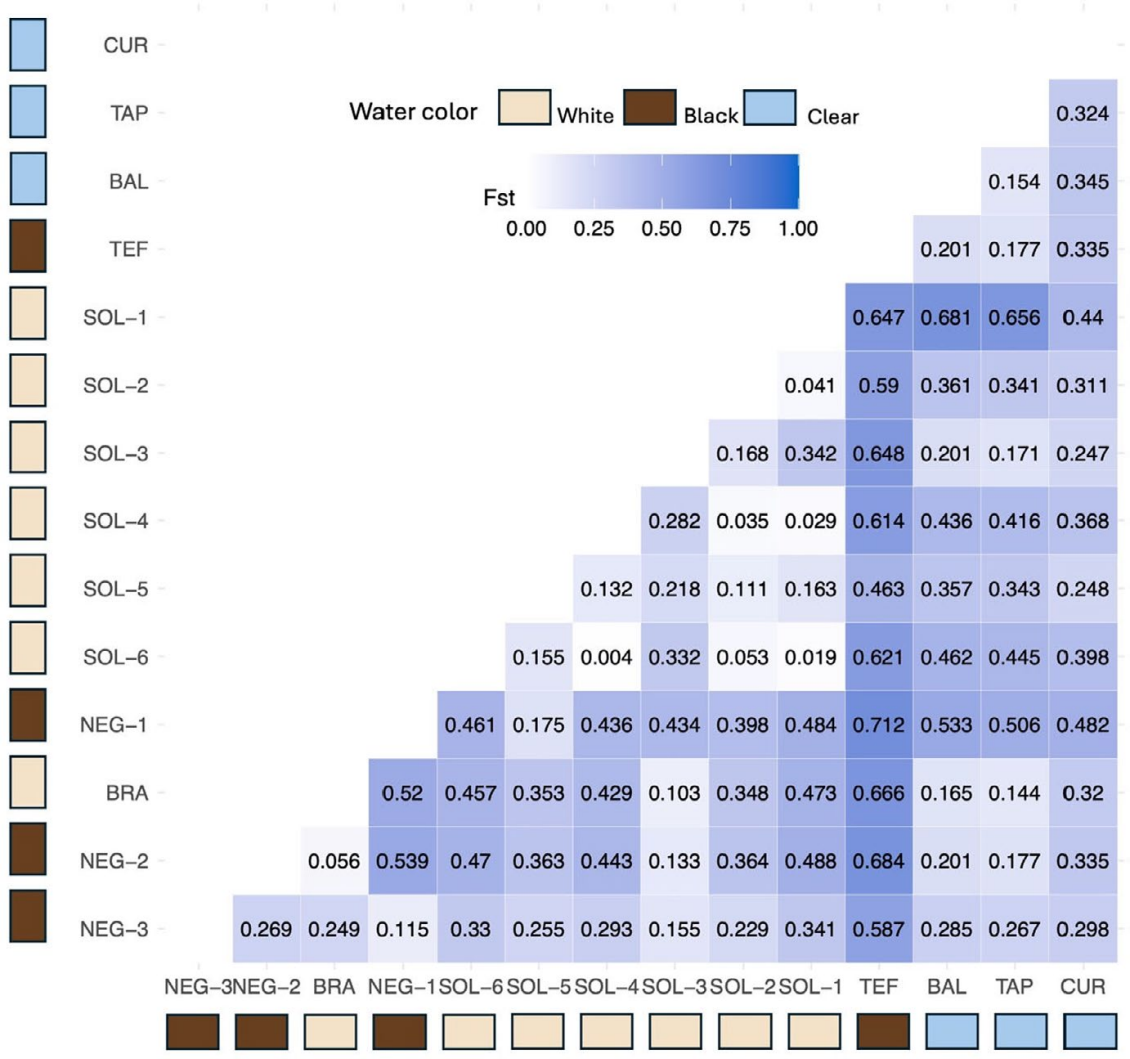
Heatmap showing fixation index (*F*_ST_) values between different sites in Amazonia. The *F*_ST_ value varies between 0 and 1, where 1 indicates complete differentiation and 0 no differentiation. The figure was produced with the *ggplot2* R package from values obtained by *Hierfstat* package in R.

The average *F*_ST_ value was 0.35 and increased slightly to 0.36 after correcting for bias (*F*_STP_) (unequal population sizes) and ranged from 0.004 (between SOL‐6 and SOL‐4) to 0.712 (between TEF and NEG‐1) (Figure 2). The TEF site showed a particularly strong differentiation with all sites, even those geographically close, such as SOL‐2 site (Figure 1, Figure 2). The *F*_ST_ value of 0.004 between groups SOL‐6 and SOL‐4 also indicates an absence of differentiation between these groups and a high gene flow (Nm = 65.79) (Table 2). In general, the lowest *F*_ST_ values and highest gene flow (Nm) values are those found between the Rio Solimões sites (Figure 2). The *F*_ST_ between Rio Solimões and Rio Negro, which presented a value of 0.12, showed moderate differentiation. The mean value of gene flow (Nm) was 1.77 (Table 2). Most sites showed low gene flow with other sites (Table 2) and the highest gene flow was found within the Rio Solimões.

**TABLE 2:** Gene flow (Nm) calculated between sites according to the formula [(**1**/***F***_**st**_) − **1]/4**.

	NEG‐3	NEG‐2	BRA	NEG‐1	SOL‐6	SOL‐5	SOL‐4	SOL‐3	SOL‐2	SOL‐1	TEF	BAL	TAP	CUR
NEG‐3		0.68	0.75	1.93	0.51	0.73	0.6	1.36	0.84	0.48	0.18	0.63	0.69	0.59
NEG‐2			4.18	0.21	0.28	0.44	0.31	1.63	0.44	0.26	0.12	0.99	1.16	0.5
BRA				0.23	0.3	0.46	0.33	2.19	0.47	0.28	0.13	1.27	1.49	0.53
NEG‐1					0.29	1.18	0.32	0.33	0.38	0.27	0.1	0.22	0.24	0.27
SOL‐6						1.37	65.79	0.5	4.49	13.1	0.15	0.29	0.31	0.38
SOL‐5							1.64	0.9	2.01	1.29	0.29	0.45	0.48	0.76
SOL‐4								0.64	6.84	8.44	0.16	0.32	0.35	0.43
SOL‐3									1.24	0.48	0.14	1	1.21	0.76
SOL‐2										5.81	0.17	0.44	0.48	0.55
SOL‐1											0.14	0.12	0.13	0.32
TEF												0.99	1.16	0.5
BAL													1.37	0.47
TAP														0.52

### 3.3 Population Structure

**FIGURE 3:**
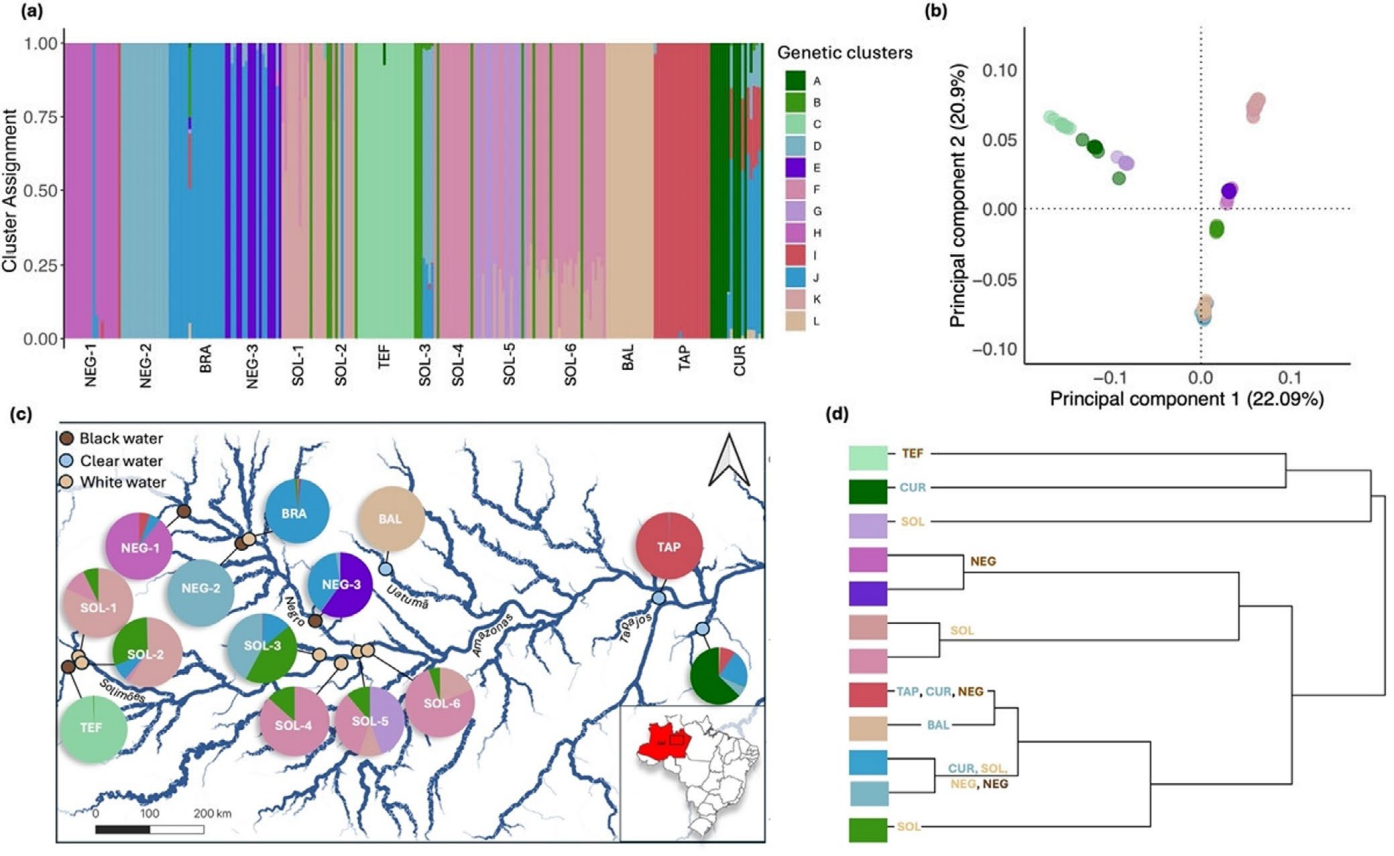
(a) Cluster assignment for individuals for *k* = 12 according to the ADMIXTURE analyses. Each bar represents an individual and individuals are grouped according to their sampling location. The colors represent their cluster assignments. Different individuals with the same color belong to the same genetic cluster. (b) Principal component analysis (PCA) calculated with *VCFtools*. The colors correspond to the different genetic clusters defined during the ADMIXTURE analyses. (c) Map of sampling sites with diagrams representing the proportions of the different genetic clusters found within the sites for *k* = 12. (d) Dendrogram of genetic cluster relationships according to the *F*_ST_ values generated by ADMIXTURE analyses.

Analysis with ADMIXTURE software showed that the most likely number of genetic clusters was 12. The smallest cv values were 0.1603 (*k* = 12) and 0.1605 (*k* = 9) (Figure S1). In the *k* = 12 groups, the Balbina site (BAL) formed a different genetic cluster, whereas for *k* = 9, individuals from the BAL site belonged to the same genetic cluster as individuals from the TAP population (Figures 3a and S2). Similarly, at *k* = 9 the NEG‐2 site and BRA sites belonged to the same genetic cluster, whereas they formed different clusters with *k* = 12. Moreover, individuals from NEG‐1 site were not included in the same genetic clusters as NEG‐3. In general, at *k* = 12, there was notably more structuring within Rio Negro than at *k* = 9. We continued the analysis with *k* = 12 groups due to its lower cv value and a more detailed and robust representation of the studied populations.

Figure 3a,c shows that some sites were associated exclusively with specific genetic clusters, such as the *L* group found exclusively at Balbina (BAL), *C* at Tefé (TEF), *G* at Janauari (SOL‐5), *H* at Barcelos (NEG‐1), and *E* at Anavilhanas (NEG‐3). Conversely, some clusters were found at several sites. For example, cluster *B* was found at all Rio Solimões sites, including sites that are very distant from one another (around 620.7 km between SOL‐1 and SOL‐6). In the PCA, we can distinguish five distinct clusters: (i) groups *C*, *A* and *G* (ii) group *B* (iii) groups *E* and *H* (iv) groups *D*, *J*, *I*, *L*, and (v) groups *F* and *K* (Figure 3b). A similar clustering was observed on the dendrogram (Figure 3d).

*F*_ST_ values between genetic groups ranged from 0.07 (between *D* and *J*) to 0.74 (between *H* and *A*). The genetics groups *K* and *F*, had a *F*_ST_ value of 0.08, showing indeed little differentiation between them (Table 3). Groups *D* and *J* had the highest gene flow (Nm = 3.08). Overall, gene flow between the different genetic groups was extremely low, ranging from 0.08 to 3.08, averaging 0.39. Cluster *G*, located at SOL‐5 site, had very low gene flow values (between 0.1 and 0.18) with the other clusters.

**TABLE 3:** Gene flow (nm) between genetic clusters calculated using the formula [(**1**/***F***_**st**_) − **1]/4**.

	A	B	C	D	E	F	G	H	I	J	K
B	0.15										
C	0.17	0.16									
D	0.14	0.39	0.15								
E	0.1	0.28	0.11	0.22							
F	0.10	0.42	0.19	0.32	0.3						
G	0.18	0.15	0.11	0.15	0.1	0.18					
H	0.09	0.24	0.1	0.19	1.87	0.31	0.08				
I	0.16	0.42	0.17	1.14	0.24	0.35	0.16	0.21			
J	0.15	0.4	0.16	3.08	0.23	0.34	0.15	0.2	1.39		
K	0.15	0.36	0.16	0.28	0.26	2.8	0.16	0.22	0.16	0.29	
L	0.14	0.38	0.15	1.01	0.22	0.32	0.14	0.19	1.27	1.24	0.28

The ratio *F*_ST_/km, was higher within the Rio Negro (mean = 0.00205) than within the Rio Solimões (0.00155) but the Wilcoxon–Mann–Whitney test was not significant (*W* = 31, *p*‐value = 0.343). We identified three outliers: the *F*_ST_/Km ratios between site TEF and SOL‐1, TEF and SOL‐2, SOL‐5 and SOL‐6. Notably, TEF and SOL‐2 exhibited the highest *F*_ST_/Km ratio.

### 3.4 Factors Impacting Gene Flow

**FIGURE 4:**
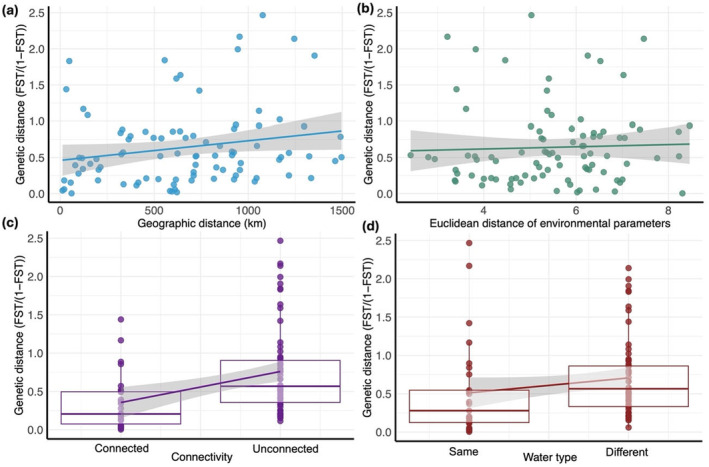
Mantel test for the genetic distance (*F*_ST_/(1 − *F*_ST_)) between sites and (a) the geographic distance (km) between sites (b) the Euclidean distance of the physicochemical parameters between sites (c) the connectivity by river flow between sites, where 0 means connected and 1 means unconnected, (d) the water type of the site where 0 means same water type and 1 means different water type.

The Mantel test was significant only for the relationship between the genetic distance and the connectivity of sites by water flow (*r* = 0.33, *p* = 0.003) and marginally significant for water type (*r* = 0.16, *p* = 0.06), but neither for the geographic distance nor for the Euclidean distance of environmental parameters (Figure 4). Furthermore, The AMOVA results showed low and non‐significant genetic variation according to water type and watershed (Table 4). However, percentage was high and significant between the sites within the same river or same water type.

**TABLE 4:** Analysis of molecular variance (AMOVA) results for site, water type, and river (only Rio Negro and Solimões in this case).

	df	Sum of squares	Percentage variation %	*p*
Water type				
Variation between water types	2	7494.603	3.54	0.18
Variation between sites within water type	12	28989.052	41.27	0.01 *
Variation within sites	233	43450.946	55.19	0.01*
Watershed				
Rivers	1	4107.134	2.78	0.20
Variation between sites within a river	9	25895.450	42.88	0.01 *
Variation within sites	181	35874.855	54.34	0.01*

The PERMANOVA was significant when the samples were grouped by water type (*R* ^2^ = 0.19, *F* = 29.5, Df = 2, *p* = 0.001) and (*R* ^2^ = 0.37, *F* = 35.42, Df = 4, *p* = 0.001). However, the *Betadisper* test was also significant for both of them (Water type = Df = 2, *R* ^2^ = 0.05, *p* = 0.003, Tributary = Df = 4, *R* ^2^ = 0.13, *p* = 0.001), showing that values among the groups are differently dispersed around their respective centroid and could have influenced PERMANOVA's significance. In addition, when coloring the PCA by water or watershed, the clusters observed did not correspond to those variables (Figure S5).

The results of the full distance‐based redundancy analysis (db‐RDA) conducted, including both AEM and dbMEM, showed significant results (*p* = 0.001) and explained 18.95% of the genetic variation (*R*_adj_^2^ = 0.1895). The first two axes accounted, respectively, for 8.12% and 5.63%. The forward selection approach revealed that dbMEM1, AEM1, AEM7, AEM6, AEM3, AEM4, AEM9, AEM8, AEM10, and AEM11 played the most important roles and were shown to be significant by the ANOVA (*p* = 0.002). The most important AEM, AEM1 (Figure S6b), showed connectivity at a high scale, separating the Solimões River from the other Amazon river tributary, the Rio Negro, but with stronger connectivity among sites near the confluence of the Rio Negro and the Rio Solimões. This AEM1 also showed highly similar values between sites within the Rio Negro (Figure S6b). Then, the second most significant AEM, AEM7, at finer spatial scales, described a separation of BAL site (Figure S6c). The partial db‐RDAs conducted were both significant. The first partial db‐RDA, conducted using dbMEMs variables while controlling for AEM, explained only 4.40% of the genetic variation (*R*_adj_^2^ = 0.0440, *p* = 0.000999), with dbMEM1 and dbMEM2 identified as significant contributors (*p* = 0.002). The first two axes accounted respectively for 3.61% and 1.76%. The partial db‐RDA conducted using AEMs while controlling for dbMEMs showed 17.10% of genetic variation (*R*_adj_^2^ = 0.1710, *p* = 0.000999), with AEM1, AEM7, AEM6, AEM3, AEM4, AEM2, AEM10, AEM8, AEM11, and AEM12 showing significant contributions (*p* = 0.002). The first two axes accounted respectively for 6.23% and 5.35%.

## 4 Species Delimitation

**FIGURE 5:**
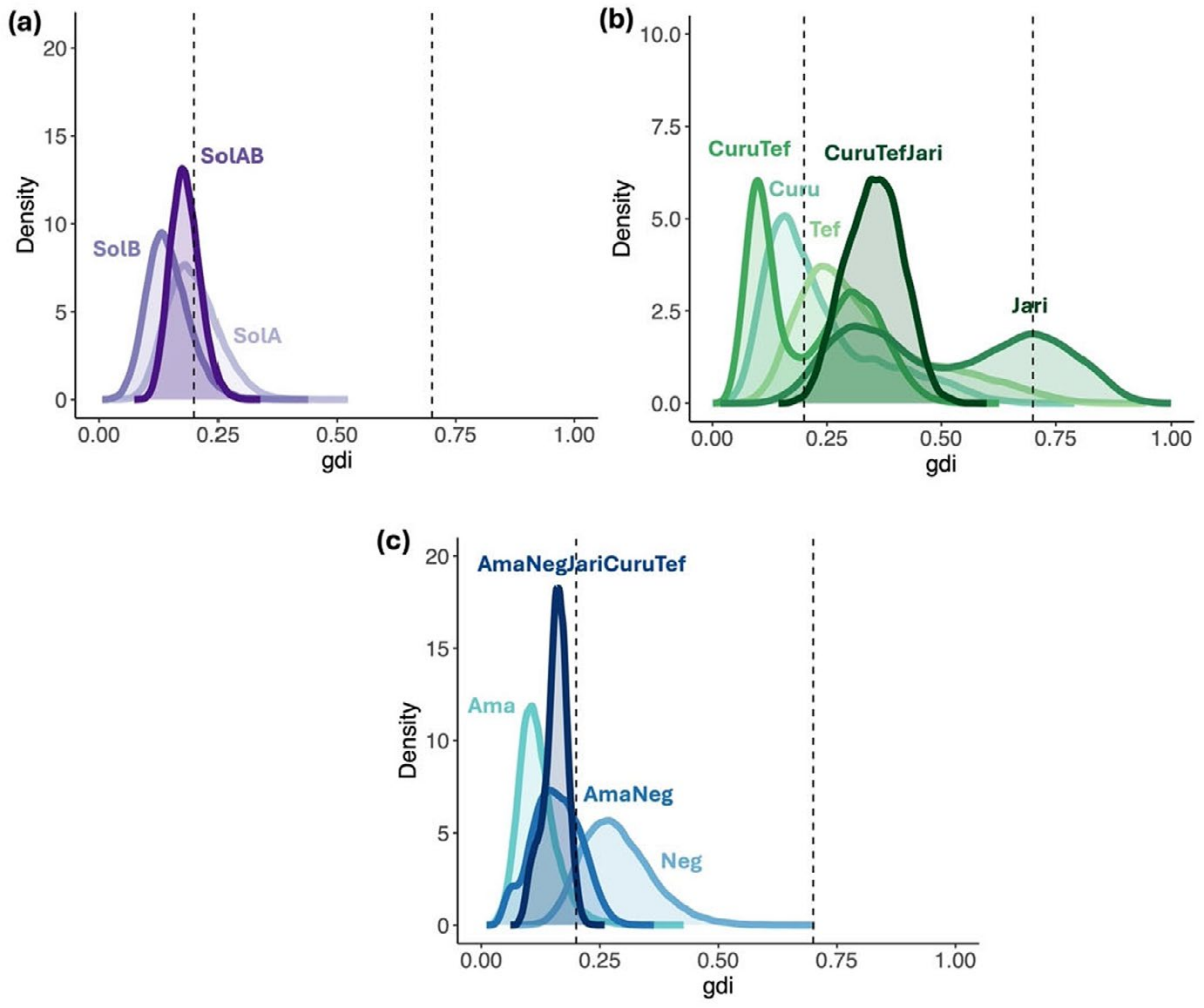
*Gdi* (*Genealogical divergence index*) values for candidate groups of putative cryptic species of *Serrasalmus rhombeus* for (a) *SolA* (formed by genetic cluster *F* and *K*), *SolB* (formed by genetic cluster *B*), *SolAB* (formed by genetic clusters *F*, *K* and *B*), (b) *Curu* (formed by genetic cluster *A*), *Tef* (formed by genetic cluster *C*) and *Jari* (formed by genetic cluster G), *CuruTef* (formed by genetic clusters *A* and *C*), *CuruTefJari* (formed by genetic clusters *A*, *C*, *G*) (c) *Ama* (formed by genetic clusters *I*, *J*, *L*, *D*), *Neg* (formed by genetic clusters *H* and *E*), *AmaNeg* (formed by genetic clusters *I*, *J*, *L*, *D*, *E*, *H*) and *AmaNegJariCuruTef* (formed by genetic clusters *I*, *J*, *L*, *D*, *E*, *H*, *A*, *C*, *G*). A *gdi* value above 0.7 suggests that the group is a distinct species, while a value below 0.2 suggests that the group is not a distinct species. Between these two values, the situation is described as ambiguous (Jackson et al. 2017).

According to PCA (Figure 3b), *F*_ST_ (Figure S3) and the maximum likelihood phylogenetic tree results (Figure S4), seven candidate groups for cryptic species emerged: *Tef* (individuals from genetic cluster C), *Curu* (individuals from genetic cluster A), *Jari* (individuals from genetic cluster G), *Neg* (individuals from genetic clusters H and E), *SolA* (individuals from genetic clusters *F* and *K*), *SolB* (individuals from genetic cluster *B*), and *Ama* (individuals from genetic clusters *I*, *J*, *L*, *D*). The *gdi* results (Figure 5) showed that genetic group *G* in *Jari* site could represent a distinct species as it exhibited a density peak above 0.7 (i.e., *gdi* threshold for putative species, see methods). No other genetic groups showed any *gdi* density peak equal or greater 0.7. However, three other genetic groups exhibited *gdi* values qualified as ambiguous (between 0.2 and 0.7), therefore providing a low to moderate signal of speciation. This was the case for group *C* in *Tef* site, as it showed *gdi* density peak at around 0.25. Then, in the group combining individuals from *Curu*, *Tef* and *Jari* sites, genetic groups A, C, and G taken together had *gdi* density peak of 0.35. Finally, genetic groups *H* and *E* from *Neg* site, exhibited a *gdi* density peak at around 0.27.

## 5 Discussion

Our study aimed to investigate the genetic diversity, population structure, and factors influencing gene flow within the black piranha ( *S. rhombeus* ) populations in central Amazonia. In addition, we explored the possible occurrence of cryptic species among the distinct genetic groups. Understanding these aspects is crucial for conservation and management efforts as well as enhancing our comprehension of the evolutionary processes and population history of this keystone species. Overall, our results showed low intra‐population genetic diversity and inter‐population gene flow, coupled with elevated levels of inbreeding, population differentiation, and clustering. In addition, we found a significant correlation between genetic distance and site connectivity by water flow, while no significant correlation was found for geographical distance, water type, or physicochemical parameters. Furthermore, our *genealogical divergence index* (*gdi*) analysis showed that the *Jari* group, located at site SOL‐5, had values above the threshold indicating the potential presence of a distinct species.

### 5.1 Genetic Diversity

The results showed a strong genetic differentiation between and within the different sites and a low genetic diversity. Specifically, the site with the highest genetic diversity was Catalão (SOL‐6). This is not surprising, given that the area studied constitutes a point of convergence between the Rio Negro and the Rio Solimões, considered an important crossroad and passage point for fish in Amazonia (Teixeira et al. 2006). The average expected heterozygosity (*H*_S_), higher than the observed heterozygosity (*H*_O_), suggested either an excess of homozygotes among the samples or a deficit of heterozygotes, as previously found for *S. rhombeus* in central Amazonia (Teixeira et al. 2006). This observation may be explained by the Wahlund effect (Wahlund 1928): when a population consists of multiple genetically distinct subpopulations with restricted gene flow between them, the occurrence of heterozygotes is lower than expected compared to scenarios where these subpopulations experience higher levels of gene flow. In contrast, sites presenting only one genetic cluster (BAL, NEG‐2, TEF, TAP) showed very low difference between *H*_S_ and *H*_O_ (BAL, TAP, TEF) (Table 1) and even a *H*_O_ value higher than *H*_S_ (NEG‐2).

Several factors contribute to low genetic diversity. These include the founder effect, where a new population originates from a small founding group, and small effective population sizes that promote genetic drift, hindering the accumulation of new mutations. Other contributing factors include the limited genetic diversity within the founding population, deviations in sex ratios, active selection by fishermen for specific traits, and isolation (Amos and Harwood 1998; Cheng et al. 2021; Piorski et al. 2008; Schmitt and Seitz 2004; Schultz et al. 2009). Furthermore, life history traits of the species can have important role on the genetic diversity (Martinez, Willoughby, and Christie 2018). Indeed, as highlighted by Hubert et al. (2007a), the larvae and juveniles of *S. rhombeus* remain in the flooded forest rather than migrating to the main channel, which limits their dispersal. Such restricted dispersion in early states of life plays a crucial role in shaping gene flow and consequently impacts on the genetic diversity and population connectivity of fishes (Baek et al. 2018).

The low diversity observed was accompanied by a low gene flow between sites and even between genetic groups, which could have reduced the genetic diversity by preventing the introduction of new genetic variants. High inbreeding over several generations can also have a considerable effect on genetic diversity by reducing the effective population size, as well as the frequency of possible genome recombination, which contributes to increased genetic isolation between individuals (review by Charlesworth 2003). The mean *F*_IS_ value of 0.20 and *F*_IT_ value of 0.53 showed a salient signature of inbreeding signal across the study sites, with nearly half showing values indicative of inbreeding (Table 1) (Wright 1949). On one hand, such inbreeding can potentially lead to a decrease in fitness and more susceptibility to diseases and environmental changes and ultimately to population extinction (Frankham 1996). However, inbreeding and low diversity effects vary greatly among species, and some are able to thrive with it (Milot et al. 2007; Schmitt and Seitz 2004; Thünken et al. 2007; Westbury et al. 2019). Similarly, genetic diversity varies between species due to their biological and ecological traits and historical backgrounds (Romiguier et al. 2014). Consequently, the low diversity found in this study could represent a characteristic of *S. rhombeus* .

As matter of fact, the low genetic diversity detected in our study is consistent with a previous study carried out on *S. rhombeus* in the Madeira basin, using intron length polymorphism (Hubert et al. 2006), with a total *H*_S_ varying between 0.15 and 0.19 and a total Ho varying between 0.12 and 0.15. In another study carried out on *S. rhombeus* (Hubert et al. 2007a), also using intron length polymorphism in the same previous location but with more sites and individuals, the results also showed a high structure but with lower differentiation than in our results, with an *F*_ST_ value varying between 0.004 and 0.207. The *H*_S_ and *H*_O_ values were also higher, ranging from 0.27 to 0.37 and 0.15 to 0.30, respectively. Similar values were found in the study by Delord et al. (2023) measuring the population genetics of different Neotropical fish species, using SNPs. Their genetic diversity values ranged between 0.358 and 0.374 for *H*_S_ and between 0.343 and 0.367 for *H*_O_. However, they found very low *F*_ST_ values, with a mean of 0.006 and a mean *F*_IS_ value of 0.029 and *F*_IT_ of 0.035. Even with sites at distances comparable to Delord et al. (2023) sampling, we did not measure such low values for *F*_ST_ and *F*_IS_ (Table 1, Figure 2). A difference in genetic differentiation pattern and structure in populations of the same species has also been found in other species of the Serrasalmidae family. For example, tambaqui ( *Colossoma macropomum* ) populations in the Rio Amazonas exhibited low values of genetic differentiation (Santos, Ruffino, and Farias 2007), while populations from the main Amazon basin and Bolivian sub‐basin showed higher values (Farias et al. 2010). Santos et al. (2016) proposed that the populations of Characiforms (Piranhas' order) are particularly prone to higher genetic differentiation whenever there are barriers or poor connectivity that disrupt gene flow. This hypothesis could explain such intraspecific differences in terms of genetic diversity observed from one site to another.

### 5.2 Black Piranha Gene Flow and Influences

For non‐migratory fish, such as *S. rhombeus* , whose dispersal capacity is estimated at around 100 km (Diaz‐Sarmiento and Alvarez‐Léon 2003), genetic structure is thought to be dependent on geographical distance (Piorski et al. 2008). Indeed, Hubert et al. (2007a) identified significant relationship between genetic distance and geographical distance between rivers (with distances ranging from 510 to 1650 km) and identified high genetic structure for sites within rivers at very low distances (10 km). The high geographical coverage of the present study (ranging from 12.5 to 1496 km between sampling sites) allowed to detect on one hand high differentiation at low distances for some sites, like TEF and SOL‐2,(distance of 32 km), and even for sympatric genetic groups, like J and D. On the other hand, strikingly, individuals from shared genetics groups (*J* and *k*) were detected along a 620 km stretch in the Rio Solimoes, translating into low *F*_ST_ values between the most distant sites: site SOL‐1 and SOL‐6. In this context, the partial db‐RDA revealed a significant but very low effect of geographical distance, explaining only 4.40% of the genetic variation, while the Mantel tests were not significant. These findings suggest that the influence of geographical distance on genetic differentiation is minimal. Besides, the population structure results indicate that *S. rhombeus* may have a greater dispersal capacity than previously assumed.

In addition, no effect of physiochemical parameters on gene flow was observed, as demonstrated by the Mantel (Figure 4) and AMOVA (Table 4) tests. This was further supported by the PCA, which showed no apparent clustering by water type. The significance of the PERMANOVA test might be attributed to differences in point dispersion around their centroid rather than differences in centroids themselves, as indicated by the betadisper test. This suggests caution regarding the reliability of the PERMANOVA performed. On the other hand, the mantel test (Figure 4d) significance could be a result of a confounding effect with the connectivity (Figure 4c), as some individuals in the same river (connected) also belong to the same water type. Still, the overall genetic structure of *S. rhombeus* populations characterized in this study remains somewhat different from what has been shown in the same region with different species (Cooke, Chao, and Beheregaray 2012a, 2012b, 2012c; Cooke, Landguth, and Beheregaray 2014). Hubert et al. (2007a) also showed no significant effect of environment on *S. rhombeus* population structure. The difference could be attributed to the fact that species in the Amazon River do not present the same tolerance for the harsh and contrasted environment of black water, and therefore, the black piranha could be less impacted by the different water types than other species. Furthermore, our sampling design had a much wider geographic coverage than previous studies, with more black and white water confluences. As highlighted by Leroux et al. (2022), such extensive sampling can reveal patterns that smaller studies with fewer black and white water confluences might have missed. Indeed, using the same sampling design as the current study, Leroux et al. (2022) also demonstrated a weak effect of water type on the population genetics of flag cichlid ( *Mesonauta festivus* ).

Finally, the only significant relationship for the mantel test was between genetic distance and sites connectivity by water flow, supported by our partial db‐RDA results, with AEMs explaining 17.10% of genomic variation. Such results have also been found by Delord et al. (2023), suggesting that *S. rhombeus* gene flow is more dependent on river flow and connectivity than on distance between sites. However, a cautious interpretation is warranted, as many pairs of sites not connected by upstream flows throughout the year actually presented a similar *F*_ST_ value to pairs that were connected (Figure 4). Overall, the *r* value for genetic distance and site connectivity from the Mantel tests, along with the *R*_adj_^2^ from the partial db‐RDA, was quite low (*r* = 0.33 and *R* _adj_^2^ = 0.1710). Furthermore, some genetics groups (*J* and *D*) are present in the two rivers (Figure 3c). Then, AMOVA showed no significant variation in terms of gene flow due to the watershed, nor did the PCA for clustering according to watershed (Figure S5). To that respect, both PCA and dendrogram (Figure 3b,d) showed that certain genetic groups are more closely related to those in different watersheds than to those in the same watershed or geographically close. For example, groups *E* and *H* of the Rio Negro are closer to groups *F* and *K* of the Rio Solimões than to groups *J* and *D* of the Rio Negro, which are geographically closer (Figure 3b,d). Also, the genetics groups *C* (TEF), *G* (SOL‐5), and *A* (CUR) formed a monophyletic group and were more closely related to each other than to other geographically close sites (Figure 3). Moreover, these three genetic groups were found in three different water types and different watersheds. Cases where individuals from different watersheds are genetically closer than individuals within the same watershed have already been previously observed in the Amazon (Lovejoy and De‐Araújo 2000; Farias et al. 2019) and may therefore suggest a historical gene flow.

### 5.3 Historical Influence

In vertebrates, cases where neither IBD nor IBE have a significant effect, but where the population have a high *F*_ST_, may suggest the presence of historical influences (such as climatic fluctuations (Hubert et al. 2007a), geological events (Sivasundar, Bermingham, and Ortí 2001), drastic population size diminution (Turner et al. 2004; Sexton, Hangartner, and Hoffmann 2014). Effect of historical factors for *S. rhombeus* has already been mentioned (Hubert et al. 2007a), as for other South American fish (Piorski et al. 2008; De Queiroz et al. 2017). However, little is known about *S. rhombeus* establishment. Hubert et al. (2007b) suggested that *S. rhombeus* started to colonize the Amazon River from east to west and the colonization of the upper course of the Amazon occurred around 3.02 ± 0.3 Mya. Another study on *S. rhombeus* reported the signature of another dispersal event in the upper Madeira during the Pleistocene (400000–800,000 years ago) (Hubert et al. 2007a). The relationship between genetic groups, which appears independent of the distance or water type, may suggest colonization of the rivers occurred at different times, or the physical separation of some groups due to landscape changes, favoring genetic differentiation (e.g., local adaptation and/or genetic drift). Numerous geographical barriers were formed in the Amazon basin during the Pleistocene, as a result of drastic climatic changes, and oscillations between glacial and interglacial periods accompanied by several marine incursions and regressions (Lundberg et al. 1998; Val et al. 2021). Those events led to major changes in river configuration, dynamics and the isolation of freshwater environments, considerably disturbing the availability of fish habitats (Albert and Reis 2011; Hoorn et al. 2010). Such major river shifts have been suggested to occur at the confluence of the Rio Solimões and Rio Negro during the late Neogene and Early Quaternary periods (Albert and Reis 2011). Then, given these historical changes, it is possible that the isolation and modification of freshwater habitats during the Pleistocene may have created physical barriers between populations, disrupting gene flow and leading to genetic differentiation within *S. rhombeus* , ultimately resulting in the genetic structure observed in our study.

### 5.4 Species Delimitation

In the present study, some results suggested reproductive isolation between groups living in sympatry and harboring high *F*_ST_ values. In their study, Nakayama et al. (2001) found black piranhas with different karyotypes living in sympatry, without the presence of hybrids. Such genetic differentiation and isolation could lead, in some cases to speciation. In our study, we detected one genetic group (*Jari, group G*) that showed a *gdi* value peak at around 0.7, the threshold of a putative cryptic species signature. Still, even if the value was above 0.7, the *gdi* approach by itself is not sufficient to confirm the presence of different species. Rather, it suggests the potential occurrence of a cryptic species. Interestingly, upon examination by a taxonomic specialist, some specimens at the SOL‐5 site showcased morphological distinctions and were categorized as *Serrasalmus aff. rhombeus*. These individuals belonged not only to the *Jari* group but also to the *SOL A* (which showed no *gdi* signature suggesting affiliation to a different species). These variances in morphology could be attributed to phenotypic variation between individuals. A much more integrative approach combining various phenotypic traits such as morphometry, non‐visual signals (sounds, pheromones, chemical communications), behavior and physiology would be required to establish the occurrence of a distinct species with confidence (Bickford et al. 2007).

Nonetheless, the cryptic species candidate *G* group was sampled in the Lago Janauari (SOL‐5), a site that presented other intriguing results. Indeed, this site is a meeting point between the Rio Solimões and Rio Negro fish populations (Rai and Hill 1981), so genetic diversity should be high. However, in this study, the opposite was found: this site exhibited a particularly low genetic diversity, the highest values of inbreeding, the highest number of private SNPs, and the highest percentage of non‐Hardy–Weinberg equilibrium loci. The significant divergence and minimal gene flow observed between the genetic clusters in this site could potentially result from a secondary contact event following the rupture of a geographical barrier. This inference aligns with prior findings indicating the proximity of the *G* genetic cluster to the *A* and *C* found in other rivers. Consequently, previous allopatric divergence could have gradually established reproductive isolation between the actual genetic clusters sampled in the site SOL‐5, therefore contributing to increased inbreeding and decrease of genetic diversity within the groups. Additional occurrences, such as a founder event, when a population is formed from a small subset of a larger population, could have exacerbated the process, leading to a reduction in genetic diversity due to the limited genetic pool of the founding individuals.

In addition, the Lago Tefé (TEF) also showed intriguing results. Indeed, the site had one endemic genetic group (*C*), showing the highest differentiation with all other sites, even with very close sites, like SOL‐2 (32.24 km, *F*_ST_ = 0.59) and SOL‐1 (48.28 km, *F*_ST_ = 0.65). As previously mentioned, genetic group *C* is more closely related to the group *G* (SOL‐5 site) and the *A* (CUR site) (Figure 3b,d) than with neighbor sites in the Rio Solimões. The striking genetic gap between SOL‐1 and TEF was previously observed for *Mesonauta festivus* (Cichlidae), a species having a different ecology and establishment history. The authors attributed this gap to the connectivity by upstream flow (Leroux et al. 2022). However, contrastingly to what was observed for *S. rhombeus* , no genetic gap was observed for *M. festivus* between SOL‐2 and TEF or with other sites from the Rio Solimões. Therefore, the pattern observed for *S. rhombeus* seems to be species specific. Our hypothesis to explain why the TEF site shows such a difference with other sites, even with nearby, connected sites, is that TEF may have been isolated from the others until recently, and therefore could not have mixed after secondary contact. However, given the absence of physical barriers, it would not explain the fact that group *C* was not found in other sites. As previously mentioned, the differences in water type have been shown to be a gene flow barrier in some others Amazonian species (Cooke, Chao, and Beheregaray 2012a, 2012b, 2012c; Cooke, Landguth, and Beheregaray 2014). TEF being a black water site, local adaptation might in part explain the genetic isolation of group *C*. However, we did not find any strong evidence for this pattern across the Amazon basin (Figures 3b,d, S5, 2, Table 4), with the other genetics groups being found in both water types at other sites. For example, the genetic cluster at BRA (white water) is also found in NEG‐1 (black water) and shows low differentiation with the group D found at NEG‐2 site (black water). However, we cannot exclude that these other genetic groups have a higher ability to inhabit different water types when compared to genetic group *C*. Little or absence of gene flow between genetic groups can result from local adaptation (Fraser et al. 2011). However, the number of private alleles in group *C* was low compared to what was observed in other groups (Table 1). In addition, we cannot rule out the possibility that our sampling missed other genetic groups inhabiting the TEF site, and that group *C* individuals are actually present in neighbor sites within the Rio Solimões. Still, the high *F*_ST_ values between TEF and neighbor sites are intriguing.

## 6 Conclusion and Perspectives

In conclusion, this high scale study which collected individuals from different watersheds and contrasting environments gives valuable insight into the Central Amazon populations of the black piranha ( *S. rhombeus* ), showing high clustering, low genetic diversity, elevated inbreeding values, and unexpected dispersion capacity beyond what was previously reported. The results underline the necessity to put more attention to this keystone species, as low genetic diversity and high inbreeding are generally deemed as hallmarks of endangered populations (Willoughby et al. 2015). To date, there are no restrictions on black piranha fishing. Although it is not considered as a major fisheries resource (Santos, Ferreira, and Zuanon 2009), it is a very popular recreational and sport fishing species. In addition, the severe drought from September–November 2023 lead to thousands of dead fishes, particularly in Tefé, where genetic diversity was particularly low. Such drastic events could lead to a population bottleneck and therefore, an even lower genetic diversity in the future (Allendorf 1986; England et al. 2003). In addition, it is important to mention that using *F*_ST_ to estimate gene flow has limitations, as this method relies on strong biological assumptions that are rarely met in most wild populations (Whitlock and McCauley 1999). As a result, it may not accurately reflect the migration patterns of the black piranha. Therefore, future studies on *S. rhombeus* population dynamics, establishment history, putative presence of cryptic species at the Janauari site and potential resilience to the upcoming deleterious events (i.e., higher water temperatures and lower O_2_) are essential for conservation purpose of this keystone species of the Amazon basin.

## Author Contributions

**Alizée Thomas:** conceptualization (lead), data curation (equal), formal analysis (lead), investigation (lead), methodology (lead), writing – original draft (lead), writing – review and editing (lead). **François‐Étienne Sylvain:** data curation (equal), funding acquisition (lead), writing – review and editing (equal). **Eric Normandeau:** data curation (equal), methodology (equal), resources (equal), writing – review and editing (equal). **Nicolas Leroux:** data curation (equal), writing – review and editing (equal). **Aleicia Holland:** data curation (supporting), writing – review and editing (equal). **Adalberto Luis Val:** funding acquisition (lead), project administration (lead), resources (equal), supervision (equal), writing – review and editing (equal). **Nicolas Derome:** conceptualization (equal), funding acquisition (lead), project administration (equal), resources (lead), supervision (lead), validation (equal), writing – review and editing (lead).

## Conflicts of Interest

The authors declare no conflicts of interest.

## Supporting information


**Data S1.** Supporting Information.

## Data Availability

The metadata and data sets generated and analyzed can be found at DataDryad (doi: 10.5061/dryad.p8cz8wb00).
