# Supplementary material for: Low Genetic Diversity and Complex Population Structure in Black Piranha ( Serrasalmus rhombeus ), a Key Amazonian Predator
Source: Ecol Evol. 2025 Feb 17;15(2):e70824. doi: 10.1002/ece3.70824 (PMC11831006; doi:10.1002/ece3.70824)
Supplement: Supplementary file 1 — Data S1. Supporting Information. [file ECE3-15-e70824-s001.docx]

**Supplementary material**

**Supplementary table 1:** Sampling sites information for this research project. The type of water at each site was determined by Sylvain and collaborators (2019), based on pH, dissolved organic carbon (DOC), optical characteristics of fluorescent dissolved organic matter (FDOM) and water conductivity.

| **Code** | **Site name** | **Coordinates** | **Water type** | **Watershed** | **Sampling period** | **n** |
| --- | --- | --- | --- | --- | --- | --- |
| BAL | Balbina | 1°50'55.9"S  59°34'59.5"W | Clear | Rio Uatumã | 10/2018 | 17 |
| BRA | Rio Branco | 1°19'05.7"S  61°52'34.7"W | White | Rio Branco | 10/2019 | 20 |
| CUR | Rio Curuá-Una | 2°48'19.1"S  54°17'52.2"W | Clear | Rio Curuá-Una | 11/2018 | 20 |
| NEG-1 | Barcelos | 0°50'50.8"S  62°57'40.3"W | Black | Rio Negro | 11/2018 | 20 |
| NEG-2 | Santo Alberto | 1°23'29.8"S  61°59'35.3"W | Black | Rio Negro | 10/2019 | 20 |
| NEG-3 | Anavilhanas | 2°41'46.1"S  60°46'33.3"W | Black | Rio Negro | 10/2018 | 20 |
| SOL-1 | Lago dos Piratas | 3°15'19.2"S  64°41'44.3"W | White | Rio Solimões | 11/2019 | 15 |
| SOL-2 | Lago Téfé-Solimões | 3°21'07.4"S  64°40'21.4"W | White | Rio Solimões | 11/2019 | 14 |
| SOL-3 | Rio Manacapuru | 3°16'16.9"S  60°42'03.2"W | White | Rio Solimões | 11/2018 | 7 |
| SOL-4 | Lago Janauacá | 3°23'37.5"S  60°19'52.6"W | White | Rio Solimões | 11/2018 | 16 |
| SOL-5 | Lago Janauari | 3°12'03.4"S  60°03'10.1"W | White | Rio Solimões | 10/2018 | 28 |
| SOL-6 | Lago Catalão | 3°09'56.4"S  59°54'38.4"W | White | Rio Solimões | 10/2018 | 20 |
| TEF | Lago Teéfé | 3°27'55.2"S  64°53'13.2"W | Black | Rio Solimões | 11/2019 | 17 |
| TAP | Rio Tapajós | 2°18'57.8"S  55°00'45.0"W | Clear | Rio Tapajós | 10/2019 | 20 |

**Supplementary table 2**: Measure of DOC quantity and FDOM optical characteristics of the water in which the *S. rhombeus* individuals were sampled

.

| **Site** | **DOC. Conc.** | **SAC340** | **SUVA254** | **Sbs254/365** | **% humic DOM** | **% Fulvic DOM** | **% Protein DOM** |
| --- | --- | --- | --- | --- | --- | --- | --- |
| **NEG-3** | 11.38 | 30.53 | 3.56 | 3.76 | 47.19 | 30.34 | 22.47 |
| **NEG-1** | 10.93 | 39.5 | 4.54 | 3.79 | 56.7 | 29.54 | 13.76 |
| **NEG-2** | 11.67 | 33.55 | 3.68 | 3.58 | 60.29 | 32.62 | 7.09 |
| **TEF** | 7.13 | 29.07 | 3.37 | 4 | 54.21 | 37.27 | 8.53 |
| **BAL** | 4.9 | 6.09 | 1.22 | 7.11 | 30.62 | 42.29 | 27.1 |
| **CUR** | 4.56 | 11.66 | 1.95 | 5.31 | 35.08 | 44.97 | 19.96 |
| **TAP** | 2.66 | 8.66 | 1.88 | 5 | 44.77 | 38.3 | 16.93 |
| **SOL-6** | 9.05 | 11.71 | 2.11 | 6.44 | 37.55 | 45.64 | 16.81 |
| **SOL-5** | 7.13 | 19.08 | 1.39 | 2.23 | 34.67 | 36.2 | 29.13 |
| **SOL-3** | 7.97 | 22.12 | 3.01 | 4.59 | 46.19 | 41.77 | 12.04 |
| **SOL-4** | 5.73 | 19.98 | 2.57 | 4.21 | 50.61 | 40.43 | 8.96 |
| **BRA** | 6.04 | 19.06 | 2.15 | 4.33 | 50.79 | 39.75 | 9.47 |
| **SOL-2** | 5.73 | 20.1 | 2.62 | 3.75 | 49.02 | 39.28 | 11.7 |
| **SOL-1** | 6.47 | 14.24 | 2.16 | 4.67 | 43.93 | 45.29 | 10.78 |

**Supplementary table 3:** Concentrations of free ions and nutrients in the water where the *S. rhombeus* individuals were sampled.

| **Site** | **Na^+^** | **Mg^+2^** | **K^+^** | **Ca^+2^** | **Cl^-^** | **Nitrite** | **Nitrate** | **Silicate** |
| --- | --- | --- | --- | --- | --- | --- | --- | --- |
| **NEG-3** | 1.8 | 0.26 | 0.65 | 0.08 | 0.32 | 0.09 | 4.36 | 72.55 |
| **NEG-1** | 0.46 | 0.12 | 0.42 | 0.04 | 0.11 | 0.11 | 3.2 | 64.41 |
| **NEG-2** | 0.25 | 0.09 | 0.33 | 0.49 | 1.16 | 0.1 | 2.87 | 92.32 |
| **TEF** | 0.87 | 0.19 | 0.56 | 0.82 | 0.53 | 0.08 | 4.09 | 217.19 |
| **BAL** | 0.8 | 0.14 | 0.67 | 0.03 | 0.78 | 0.05 | 1.55 | 85.93 |
| **CUR** | 1.52 | 0.26 | 0.67 | 0.04 | 1.22 | 0.06 | 2.55 | 171.96 |
| **TAP** | 0.43 | 0.47 | 0.57 | 0.68 | 0.39 | 0.09 | 1.91 | 179.36 |
| **SOL-6** | 4.56 | 3.76 | 1.71 | 0.83 | 1.75 | 0.09 | 0.56 | 242.31 |
| **SOL-5** | 1.99 | 0.2 | 0.79 | 0.06 | 1.47 | 0.19 | 1.31 | 98.31 |
| **SOL-3** | 4.91 | 0.14 | 1.45 | 0.05 | 1.43 | 0.12 | 1.53 | 126.01 |
| **SOL-4** | 3.32 | 1 | 1.07 | 0.44 | 2.17 | 0.13 | 20.45 | 156.51 |
| **BRA** | 1.15 | 0.43 | 0.7 | 0.93 | 1.1 | 0.04 | 8.23 | 180.48 |
| **SOL-2** | 1.95 | 0.21 | 0.28 | 1.11 | 1.29 | 0.03 | 6.47 | 326.53 |
| **SOL-1** | 5.35 | 1.76 | 1.28 | 1.17 | 3.26 | 0.61 | 11.96 | 222.31 |

**Supplementary table 4:**  Primary productivity characterization and measure of physicochemical parameters in the water where the *S. rhombeus* individuals were sampled.

| **Site** | **Chl a Conc.** | | **Pheopigments Conc.** | **Chla/DOC** | **Temperature**  **(**°C) | **Cond.**  **(**uS) | **pH** | **% O_2_** |
| --- | --- | --- | --- | --- | --- | --- | --- | --- |
| **NEG-3** | | 0.05 | 0.38 | 0 | 30.7 | 13.2 | 4.24 | 53.2 |
| **NEG-1** | | 0.35 | 2.43 | 0.03 | 31.6 | 13.1 | 3.71 | 92.12 |
| **NEG-2** | | 0.73 | 0.33 | 0.06 | 30.6 | 10.6 | 4.16 | 58 |
| **TEF** | | 1.82 | 1.73 | 0.26 | 30 | 10.6 | 4.98 | 61.5 |
| **BAL** | | 0.83 | 0.78 | 0.17 | 33.2 | 16.8 | 5.05 | 103.2 |
| **CUR** | | 1.25 | 2.38 | 0.28 | 31.2 | 19 | 6 | 79.1 |
| **TAP** | | 2.15 | 1.03 | 0.81 | 30 | 14.1 | 6.36 | 80.2 |
| **SOL-6** | | 7.14 | 6.6 | 0.79 | 32.9 | 174.8 | 5.7 | 44 |
| **SOL-5** | | 4.62 | 17.31 | 0.65 | 32.9 | 22.4 | 4.38 | 60 |
| **SOL-3** | | 2.78 | 10.54 | 0.35 | 32.6 | 24.3 | 5.31 | 72.8 |
| **SOL-4** | | 1.35 | 1.88 | 0.24 | 29.3 | 88 | 6.75 | 82.6 |
| **BRA** | | 6.21 | 2.89 | 1.03 | 31 | 22 | 6.25 | 88.7 |
| **SOL-2** | | 4.41 | 3.2 | 0.77 | 30.3 | 19.7 | 6.05 | 68.6 |
| **SOL-1** | | 9.05 | 4.69 | 1.4 | 31.9 | 127.6 | 7.15 | 31.9 |

**Supplementary table 5:** Concentration of dissolved metals (ug/L) in the water where the *S. rhombeus* individuals were sampled.

| **Site** | **Al** | **V** | **Cr** | **Mn** | **Fe** | **Co** | **Ni** | **Cu** | **Zn** | **As** | **Cd** | **Pb** |
| --- | --- | --- | --- | --- | --- | --- | --- | --- | --- | --- | --- | --- |
| **NEG-3** | 36.33 | 0.34 | 0.37 | 9.24 | 142.38 | 0.28 | 3.23 | 9.25 | 72.92 | 0.48 | 0.21 | 1.11 |
| **NEG-1** | 137.75 | 0.38 | 0.3 | 7.38 | 166.63 | 0.13 | 1.93 | 10.36 | 33.48 | 0.16 | 0.09 | 1.44 |
| **NEG-2** | 150 | 0.1 | 0.05 | 5.9 | 160 | 0.1 | 0.15 | 0.3 | 11 | 0.05 | 0.02 | 0.27 |
| **TEF** | 62 | 0.1 | 0.33 | 13 | 220 | 0.1 | 0.52 | 0.6 | 4.4 | 0.19 | 0.02 | 0.12 |
| **BAL** | 10.29 | 0.05 | 0.05 | 0.23 | 16.85 | 0.1 | 0.13 | 0.56 | 4.49 | 0.14 | 0.02 | 0.05 |
| **CUR** | 18.49 | 0.17 | 0.58 | 12.31 | 52.88 | 0.11 | 1.18 | 2.12 | 23.94 | 0.64 | 0.07 | 0.25 |
| **TAP** | 5 | 0.1 | 0.05 | 0.05 | 7 | 0.1 | 0.1 | 0.5 | 3.7 | 0.07 | 0.02 | 0.03 |
| **SOL-6** | 1.81 | 0.17 | 0.1 | 0.61 | 5.84 | 0.1 | 0.48 | 2.2 | 171.78 | 0.99 | 0.02 | 0.03 |
| **SOL-5** | 65.5 | 0.78 | 0.4 | 9.85 | 269.28 | 0.1 | 0.85 | 16.19 | 44.15 | 0.47 | 0.06 | 0.67 |
| **SOL-3** | 13.47 | 0.85 | 0.21 | 4.64 | 97.85 | 0.1 | 1.12 | 2.11 | 25.85 | 0.38 | 0.08 | 0.16 |
| **SOL-4** | 28.02 | 1.45 | 0.09 | 11.25 | 166.97 | 0.1 | 0.58 | 2.73 | 1.83 | 0.71 | 0.03 | 0.25 |
| **BRA** | 38 | 0.2 | 0.05 | 0.51 | 230 | 0.1 | 0.14 | 0.8 | 2.6 | 0.07 | 0.02 | 0.26 |
| **SOL-2** | 49 | 0.3 | 0.11 | 0.68 | 250 | 0.1 | 0.41 | 0.5 | 2.7 | 0.27 | 0.02 | 0.21 |
| **SOL-1** | 27 | 0.2 | 0.06 | 4.6 | 82 | 0.1 | 0.6 | 1.7 | 8.1 | 1.3 | 0.02 | 0.11 |

*
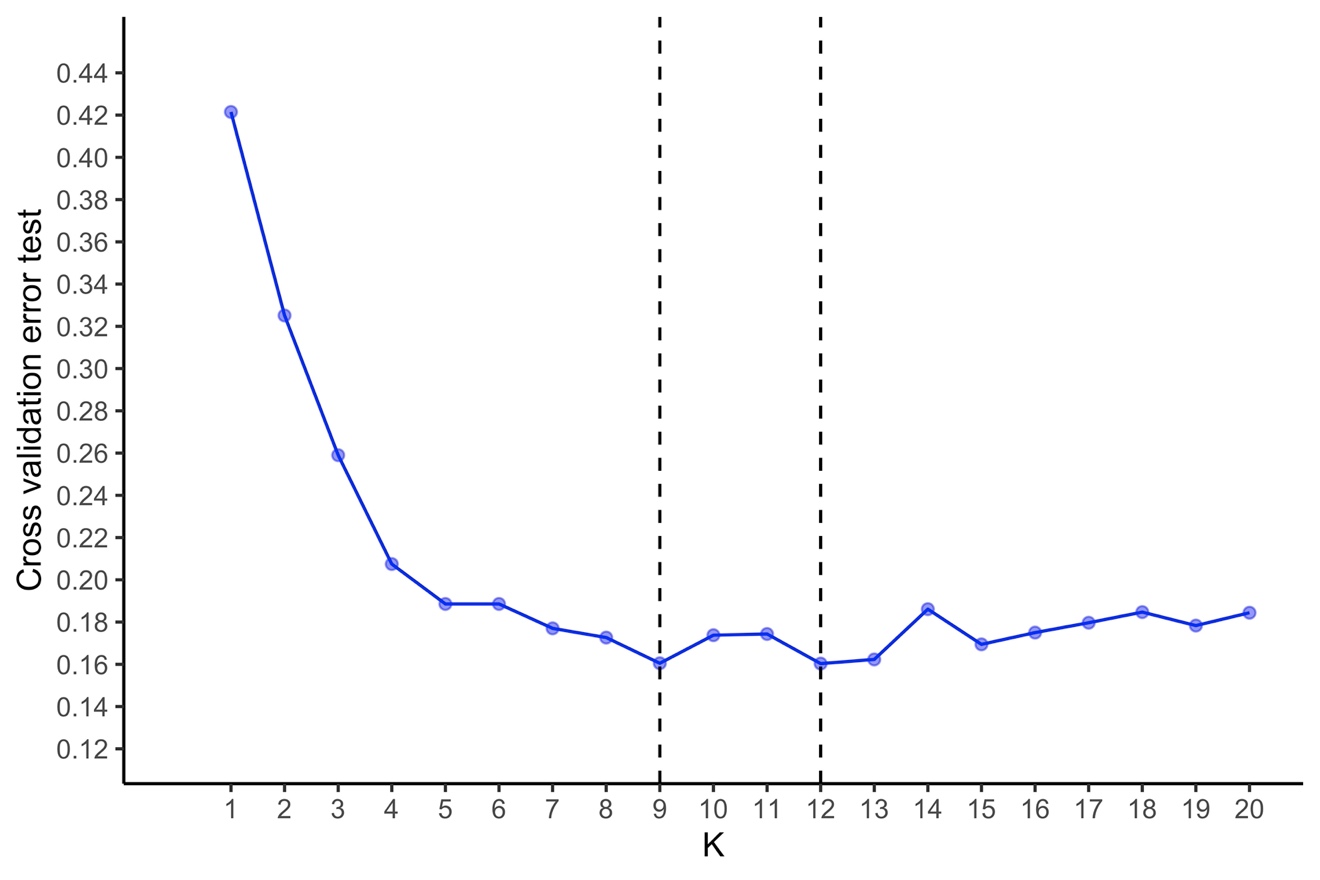
*

**Supplementary figure 1**: Cross-validation error test values (cv) according to k value. The smallest values correspond to k = 9 and k = 12. Values obtained with ADMIXTURE.

*
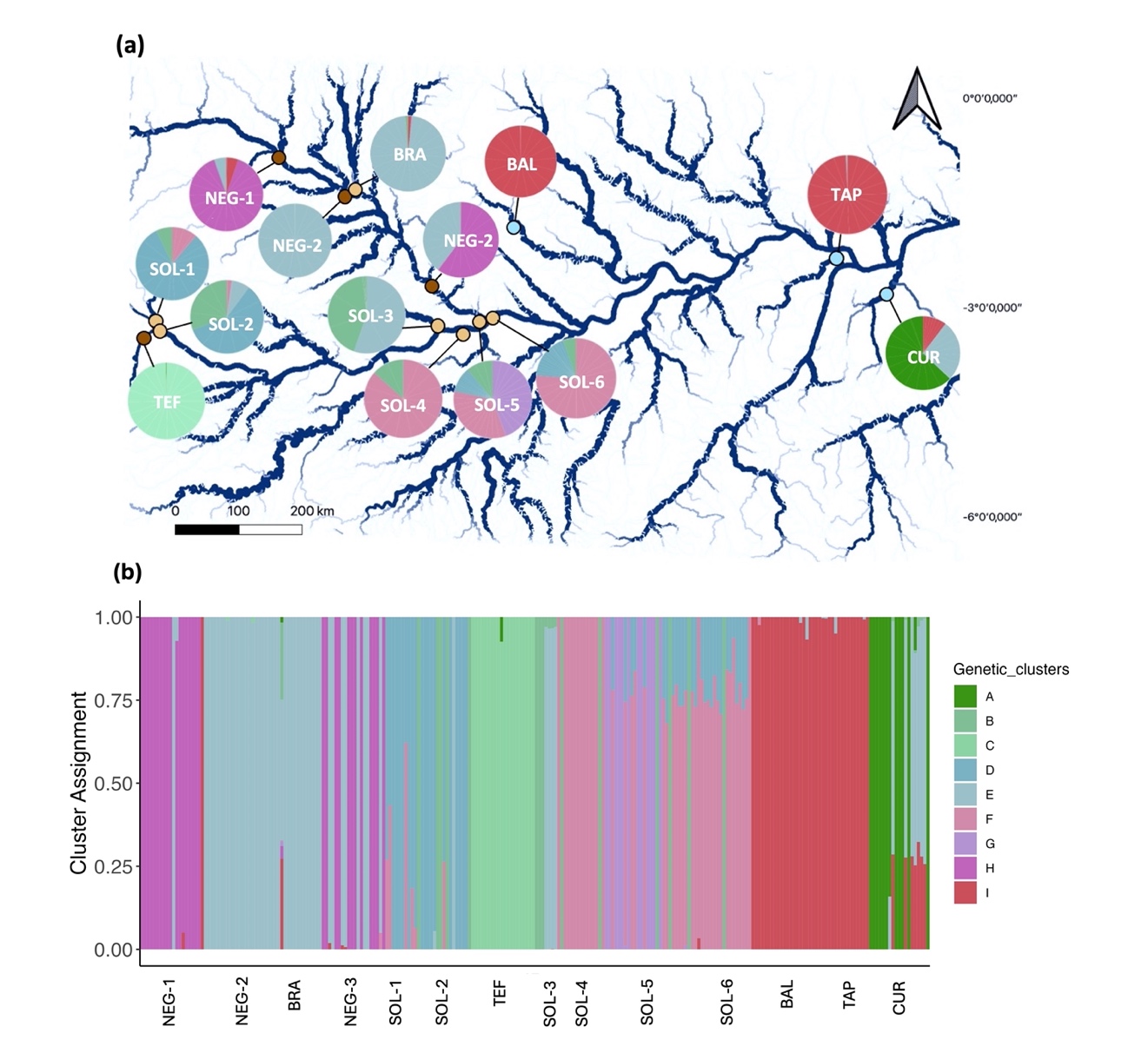
*

**Supplementary figure 2**: **(a)** Map of sampling sites with diagrams representing the proportions of different genetic clusters found within sites for k = 9. **(b)** Cluster assignment for individuals for *k* = 9.

**
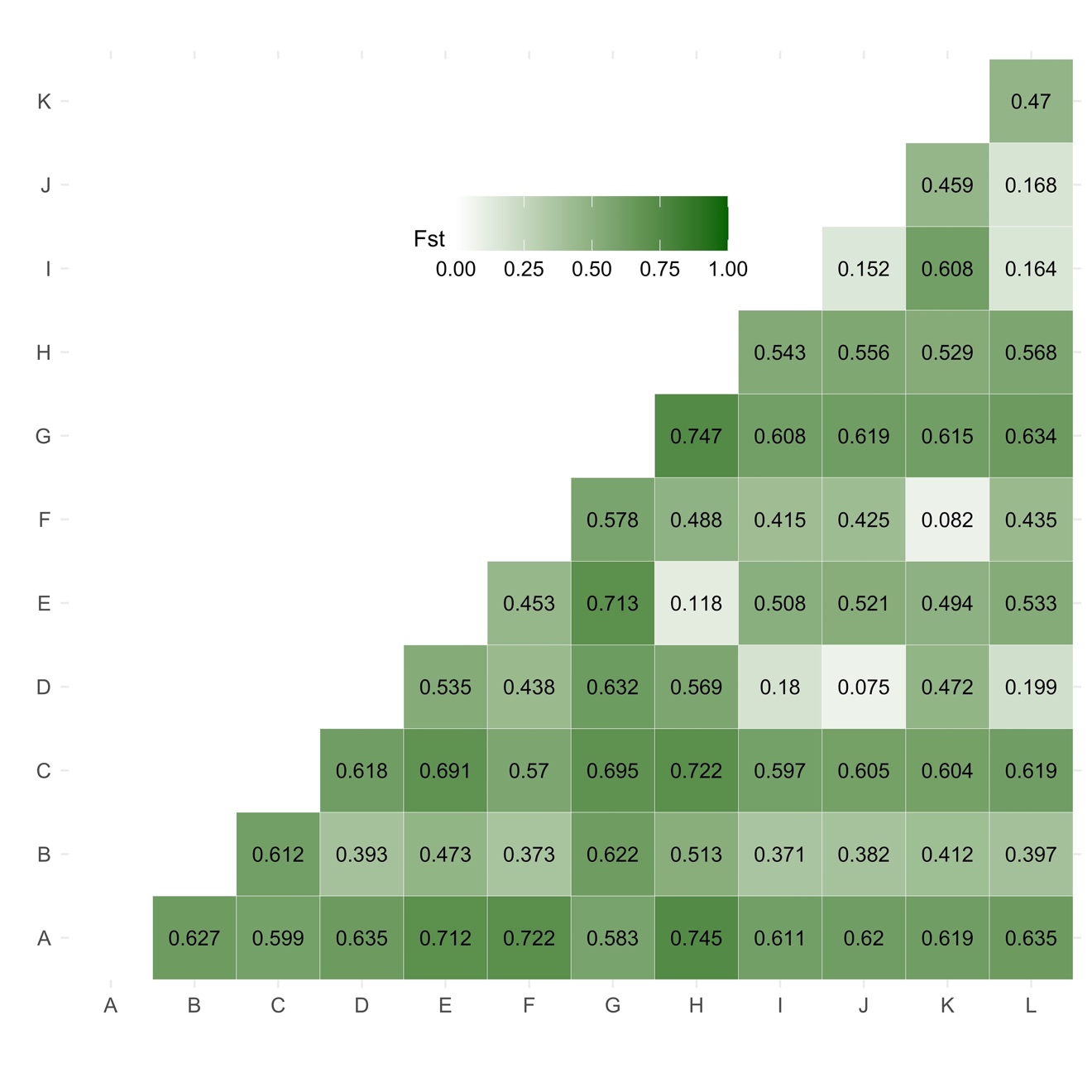
**

**Supplementary Figure 3**: Heatmap showing the values of the fixation index (F_ST_) between the different genetic clusters of black piranhas in Central Amazonia. The F_ST_ value varied between 0 and 1, where 1 indicates complete differentiation and 0 no differentiation. The higher the F_ST_ value, the greater the difference between sites. Values were obtained with ADMIXTURE software.

**
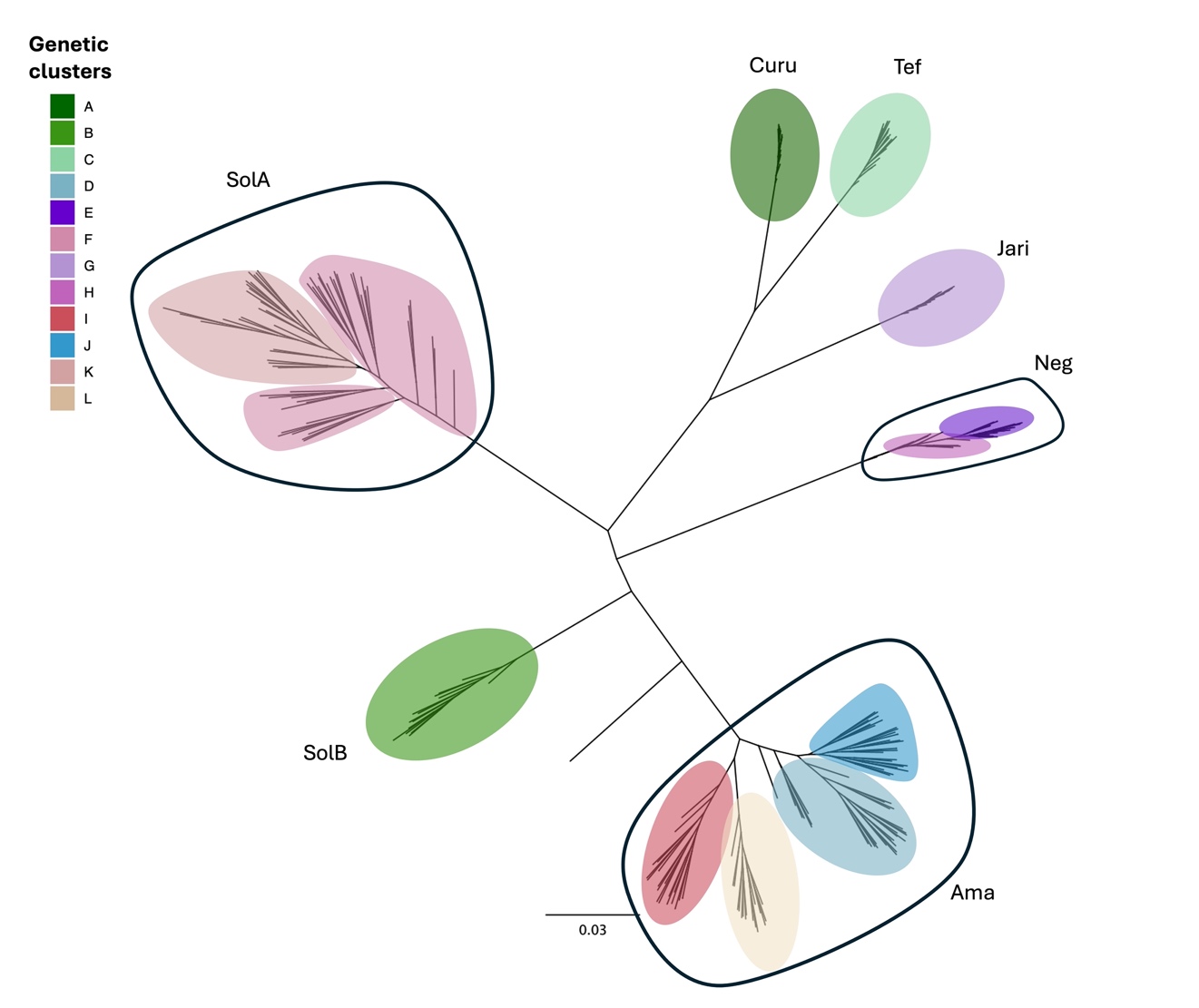
**

**Supplementary figure 4**: Maximum likelihood phylogenetic tree generated by *IQ-tree* with the TVM+F+R5+ASC model. The colors correspond to the genetic clusters generated by the ADMIXTURE analysis. The groups Jari, Tef, Cur, Ama, Neg, Sol-A, Sol-B are the groups used for the *gdi* analysis.


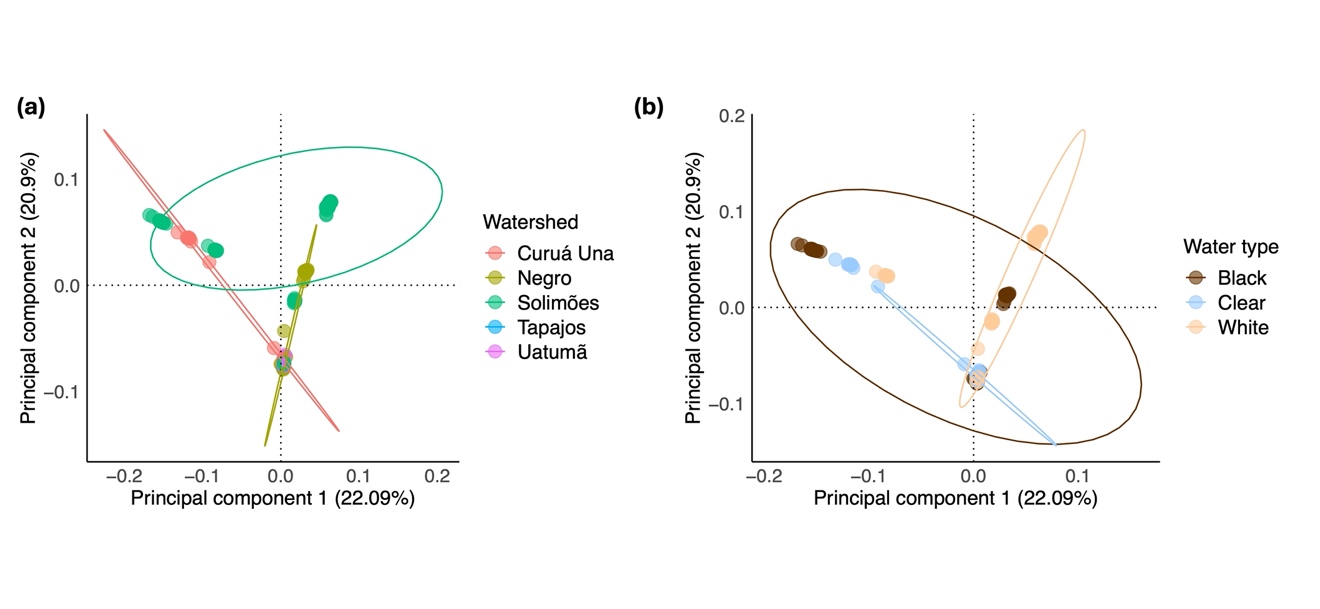


**Supplementary figure 5**: Principal component analysis with individuals colored according to **(a)** Water type **(b)** Watershed.


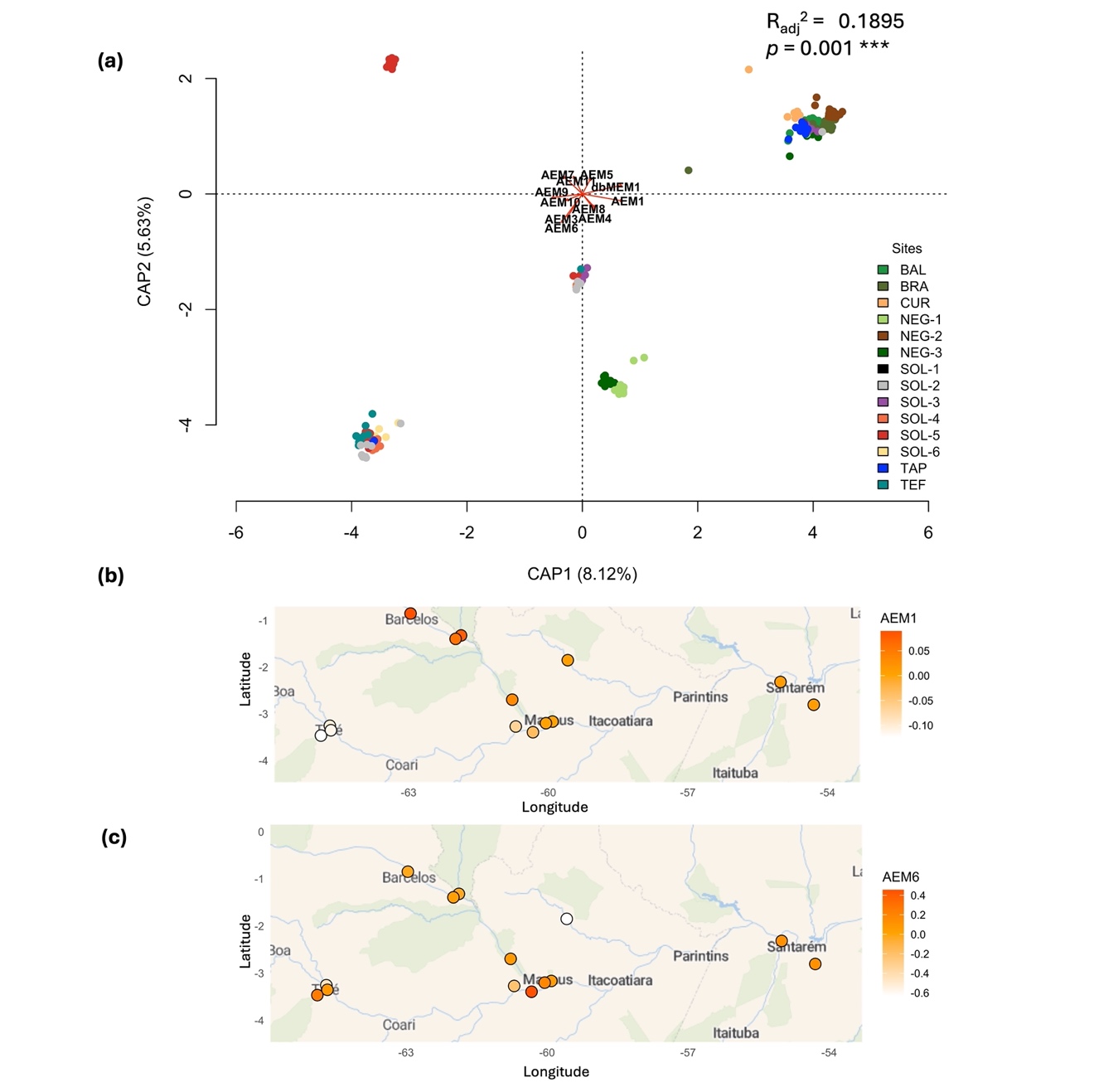


**Supplementary figure 6**: **(a)** db-RDA (distance-based redundancy analysis (db-RDA) triplot (scaling 2) showing the significant Asymmetric Eigenvector Maps (AEM) and distance-based Moran’s Eigenvector Maps (dbMEM) **(b)** most significant AEM, AEM1, showing an effect at broad scale and **(c)** second most significant AEM, AEM6, showing an effect at a finer scale.


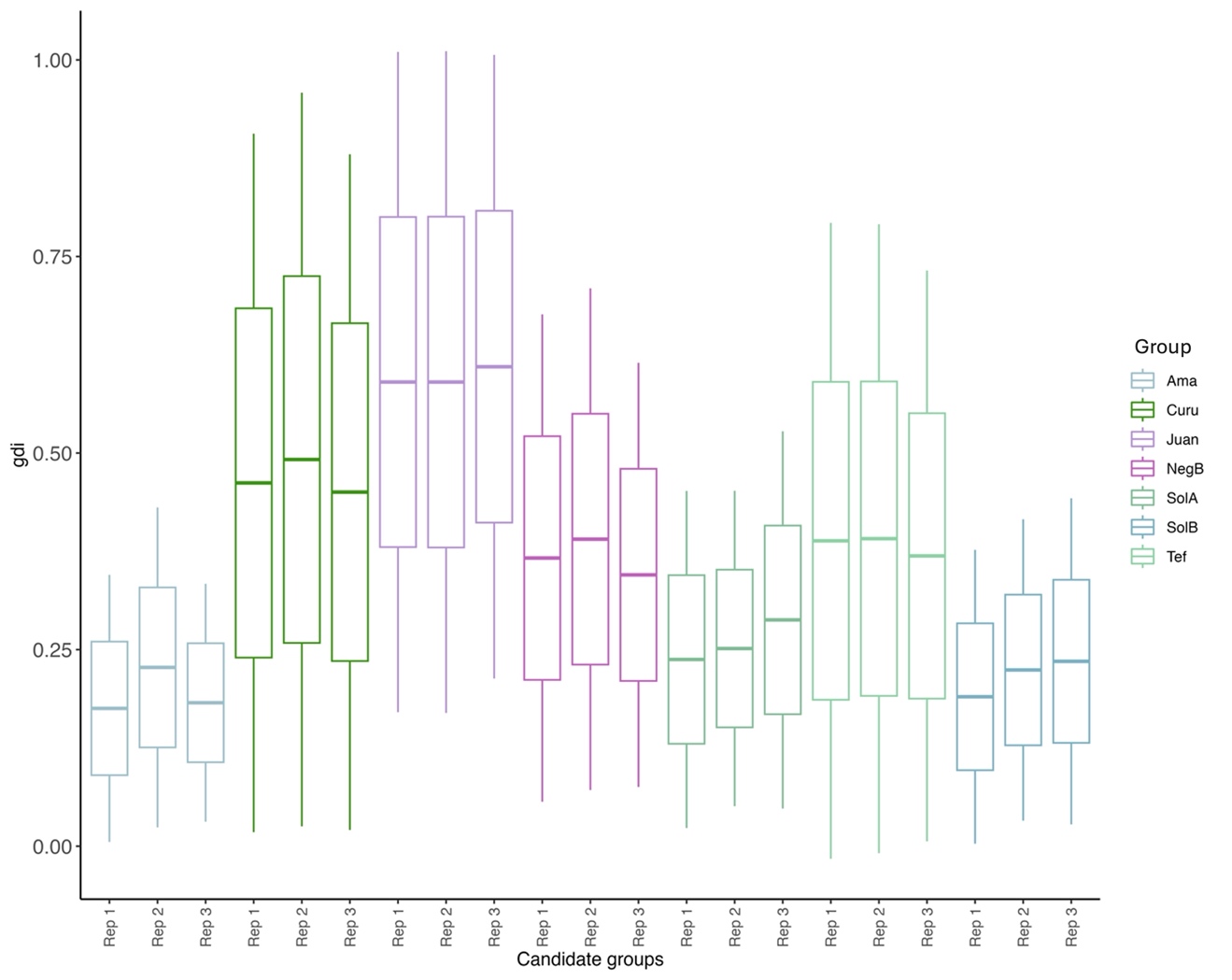


**Supplementary figure 7:** gdi values for three different sets of 700 SNPS sampled genome-wide for each group.
